# PEGylated Zein Micelles for Prostate Cancer Therapy: Influence of PEG Chain Length and Transferrin Targeting on Docetaxel Delivery

**DOI:** 10.3390/pharmaceutics18010068

**Published:** 2026-01-04

**Authors:** Khadeejah Maeyouf, Jitkasem Meewan, Hawraa Ali-Jerman, Musa Albatsh, Sukrut Somani, Partha Laskar, Margaret Mullin, Craig Irving, Graeme MacKenzie, Christine Dufès

**Affiliations:** 1 Strathclyde Institute of Pharmacy and Biomedical Sciences, University of Strathclyde, 161 Cathedral Street, Glasgow G4 0RE, UK; khadijaalmabroukalzwia@gmail.com (K.M.); jmeewan@gmail.com (J.M.); hawraa.jerman@ku.edu.kw (H.A.-J.); malbatsh@meu.edu.jo (M.A.); sukrut.somani@outlook.com (S.S.); plaskar@gitam.edu (P.L.); graeme.mackenzie@strath.ac.uk (G.M.); 2 College of Pharmacy, Health Sciences Centre, Kuwait University, Jabriya P.O. Box 12345, Kuwait; 3 Faculty of Pharmacy, Middle East University, Queen Aliaa Airport Street, Amman 11610, Jordan; 4 Department of Chemistry, School of Science, Gandhi Institute of Technology and Management, Visakhapatnam 530045, India; 5 Cell Analysis Facility, Medical and Veterinary & Life Sciences Shared Research Facilities, College of Medical, Veterinary and Life Sciences, University of Glasgow, Glasgow G12 8QQ, UK; Margaret.Mullin@glasgow.ac.uk; 6 Department of Pure and Applied Chemistry, University of Strathclyde, 295 Cathedral Street, Glasgow G1 1XL, UK; craig.irving@strath.ac.uk

**Keywords:** zein, transferrin, docetaxel, micelles, polyethylene glycol, tumor targeting, prostate cancer

## Abstract

**Background/Objectives:** Docetaxel is a widely used chemotherapeutic agent for several malignancies and is an established treatment for castration-resistant prostate cancer. However, its poor aqueous solubility, systemic toxicity, and the emergence of drug resistance limit its clinical benefit. Zein, a prolamin, forms micelles that enhance the solubility and delivery of hydrophobic drugs. As PEG length and ligand presentation govern micelle behavior, we investigated transferrin-functionalized PEGylated zein micelles as docetaxel nanocarriers and examined how PEG chain length (5 K vs. 10 K) and transferrin-mediated targeting affect delivery to prostate cancer cells. **Methods:** Docetaxel-loaded zein micelles bearing 5 K or 10 K PEG chains were prepared and conjugated to transferrin. Formulations were characterized for size, charge, morphology, critical micelle concentration, colloidal stability, drug loading and transferrin density. Cellular uptake and mechanisms were assessed in PC-3-Luc, DU145 and LNCaP cells by confocal microscopy, flow cytometry and pharmacological inhibition. Anti-proliferative activity was determined by MTT assays. **Results:** Both PEG5K and PEG10K micelles formed micellar dispersions with low polydispersity and high encapsulation efficiency. PEG5K micelles achieved higher transferrin conjugation and drug loading. Transferrin-functionalized PEG5K micelles showed enhanced uptake in DU145 and LNCaP cells but lower internalization in PC-3-Luc cells. Inhibitor studies indicated receptor-dependent uptake via clathrin- and caveolae-mediated endocytosis. Free docetaxel remained the most potent. However, among nanocarriers, transferrin-targeted PEG5K micelles showed the greatest anti-proliferative efficacy relative to their non-targeted counterparts, whereas transferrin-targeted PEG10K micelles were less potent than the non-targeted PEG10K micelles across all three cell lines. **Conclusions:** PEG chain length and ligand presentation are key determinants of uptake and cytotoxicity of docetaxel-loaded zein micelles. Shorter PEG chains favor effective transferrin display and receptor engagement, whereas longer PEG likely induces steric hindrance and reduces targeting, supporting transferrin-conjugated PEG5K zein micelles (the lead formulation in this study) as a targeted delivery platform that improves performance relative to matched non-targeted micelles in vitro, while free docetaxel remains more potent in 2D monolayer assays.

## 1. Introduction

Prostate cancer is one of the most frequently diagnosed malignancies in men worldwide, representing a major public health concern with a growing global burden. According to the Global Cancer Observatory, it was the second most commonly diagnosed malignancy in men in 2020, with over 1.4 million new cases and approximately 375,000 deaths globally, corresponding to 8% of all deaths caused by cancer in men [1]. While early-stage prostate cancer can often be managed with surgery or radiation therapy, advanced and metastatic forms require systemic treatment, including hormonal therapy and chemotherapy [2].

Docetaxel (DTX) is a semi-synthetic taxane widely used in the management of several solid malignancies (including breast, non-small cell lung, gastric, head and neck, ovarian, and prostate cancers) and remains a cornerstone of systemic therapy for advanced prostate cancer, including metastatic castration-resistant disease [3,4]. It promotes tubulin polymerization and stabilizes microtubules, thereby inhibiting mitotic cell division. Despite its clinical utility, DTX’s extremely low aqueous solubility, dose-limiting toxicities, and the emergence of drug resistance can compromise tumor exposure and therapeutic benefit [5,6].

To address these limitations, various nanocarrier-based drug delivery systems have been explored. Among them, polymeric micelles have attracted considerable attention due to their ability to improve the solubility, stability, and bioavailability of hydrophobic drugs like docetaxel, while enabling passive or active tumor targeting [7,8]. By increasing apparent aqueous dispersibility and shielding hydrophobic payloads within a core–shell structure, micelles can improve effective drug availability at the target site and help mitigate off-target exposure. These nanoscale assemblies typically consist of amphiphilic copolymers that self-assemble in water to form core–shell nanostructures, enabling drug encapsulation, protection and (potentially) altered cellular delivery profiles relative to free drug [7].

Zein, a hydrophobic prolamin derived from maize, has recently emerged as a promising natural polymer for drug delivery applications. Its unique amphiphilic structure, biocompatibility, biodegradability, and Generally Recognized As Safe (GRAS) status make it particularly suitable for formulating micellar systems [9,10,11,12,13,14]. Zein-based micelles have been shown to efficiently encapsulate a range of hydrophobic agents, including chemotherapeutics, and offer enhanced stability and sustained drug release profiles [10,15,16,17]. Zein-based nanocarriers have also been investigated for enhancing the delivery of other poorly water-soluble anticancer drugs, including docetaxel-loaded zein formulations designed to improve colloidal stability, pharmacokinetics, and antitumor efficacy. For example, chondroitin sulfate-hybridized zein nanoparticles enabled tumor-targeted docetaxel delivery and improved in vivo performance compared with free drug [18]. In addition, glucose-modified zein nanoparticles have been reported to markedly increase docetaxel oral bioavailability and antitumor activity in vivo, highlighting zein’s versatility as a carrier platform [19].

However, the hydrophobic nature of zein can limit its colloidal stability and circulation time under physiological conditions. To overcome this, surface modification with polyethylene glycol (PEG) has been widely employed. PEGylation offers multiple benefits: it improves the aqueous dispersibility of zein-based micelles and provides steric stabilization to prevent aggregation [20]. In some nanoparticle systems, sufficiently dense PEG coatings have been associated with reduced opsonization and clearance by the mononuclear phagocyte system, thereby prolonging systemic circulation [15,16,17,20,21,22,23,24]. Additionally, PEG chains on the micelle surface can serve as functional linkers for conjugating targeting ligands such as transferrin, facilitating active targeting without compromising micellar stability. Importantly, the molecular weight of PEG influences these characteristics: longer chains may offer greater steric protection but can also hinder ligand accessibility, while shorter chains provide improved targeting potential but may compromise stability [21,25].

While passive targeting through the enhanced permeability and retention (EPR) effect improves tumor accumulation to some extent, it remains insufficient for many solid tumors, including prostate cancer. Therefore, ligand-mediated active targeting strategies have been developed to enhance the selective delivery of nanocarriers to cancer cells. One such approach involves exploiting the transferrin receptor (TfR), which is overexpressed in numerous malignancies, including prostate cancer, due to the increased iron demands of rapidly proliferating cells [26,27]. Transferrin-functionalized nanocarriers can bind specifically to TfR, facilitating receptor-mediated endocytosis and intracellular drug delivery, which can enhance the therapeutic index while minimizing off-target toxicity [26,28]. Consistent with this rationale, transferrin functionalization has been explored for docetaxel delivery to prostate cancer cells, including transferrin-functionalized liposomes that enhanced interaction with TfR-expressing prostate cancer models [29,30,31,32]. Moreover, we previously reported transferrin-bearing zein-based hybrid nanoparticles for docetaxel delivery to prostate cancer cells, supporting the feasibility of combining zein and transferrin in ligand-directed taxane delivery systems [24].

In this study, we investigated the development of PEGylated zein micelles conjugated with transferrin as a targeted nanoplatform for docetaxel delivery in prostate cancer. We evaluated the impact of PEG chain length (5 K vs. 10 K) and transferrin conjugation on micellar characteristics, cellular uptake, and anti-proliferative efficacy across three prostate cancer cell lines, PC-3-Luc, DU145, and LNCaP. Our aim was to elucidate how these physicochemical and biological parameters influence the therapeutic performance of docetaxel-loaded micelles, ultimately contributing to the rational design of more effective nanomedicines for prostate cancer therapy.

## 2. Materials and Methods

### 2.1. Cell Lines and Reagents

Yellow zein (from maize), human holo-transferrin (Tf), cupric sulphate (pentahydrate) (CuSO_4_.5 H_2_O), 2-Iminothiolane hydrochloride (Traut’s reagent), dimethyl sulfoxide (DMSO), deuterated dimethyl sulfoxide-d_6_, absolute ethanol (≥99.8%), Folin and Ciocalteu’s phenol reagent, anhydrous sodium carbonate (Na_2_CO_3_), sodium potassium tartrate (C_4_H_4_KNaO_6_), bovine serum albumin (BSA), Vivaspin^®^ 6 centrifugal filter tubes with molecular weight cut-offs (MWCO) of 5000 and 100,000 Daltons, citric acid monohydrate, trisodium citrate dihydrate, docetaxel *purum* (≥97.0%), sodium hydroxide (NaOH), chlorpromazine, colchicine, filipin, poly-L-lysine, phosphate buffer saline, 3-(4,5-dimethylthiazol-2-yl)-2,5-diphenyl-tetrazolium bromide (MTT), trypsin, Nile red, Triton X-100, and Minimum Essential Eagle’s Medium (MEM) were purchased from Sigma-Aldrich (Poole, UK). Methoxy PEG succinimidyl carboxymethyl esters with molecular weights of 5000 Da (mPEG-SCM-5K) and 10,000 Da (mPEG-SCM-10K) were obtained from JenKem Technology (Plano, TX, USA). Glycine and Snakeskin^®^ dialysis tubing (7–10 kDa MWCO) were supplied by Thermo Fisher Scientific (Loughborough, UK). Sodium pyruvate, L-glutamine, penicillin-streptomycin, 4-(2-hydroxyethyl) piperazine-1-ethanesulfonic acid (HEPES buffer, 10 mM), Roswell Park Memorial Institute (RPMI)-1640 cell culture medium, fetal bovine serum (FBS), and Tubulin Tracker^®^ Green (Oregon Green^®^ 488 Taxol, bis-acetate) were purchased from Life Technologies (Paisley, UK). Vectashield^®^ mounting medium containing 4′,6-diamidino-2-phenylindole (DAPI) was purchased from Vector Laboratories (Peterborough, UK).

The Bioware^®^ PC-3M-luc-C6 human prostate adenocarcinoma cell line that expresses the firefly luciferase and is androgen-independent was obtained from Caliper Life Sciences (Hopkinton, MA, USA). The androgen-independent DU145 and androgen-sensitive LNCaP prostate cancer cell lines were sourced from the European Collection of Cell Cultures (ECACC) (Salisbury, UK).

### 2.2. Synthesis of Transferrin-Bearing PEGylated Zein-Based Micelles Loading Docetaxel

#### 2.2.1. Synthesis of PEGylated Zein

**Figure 1:**
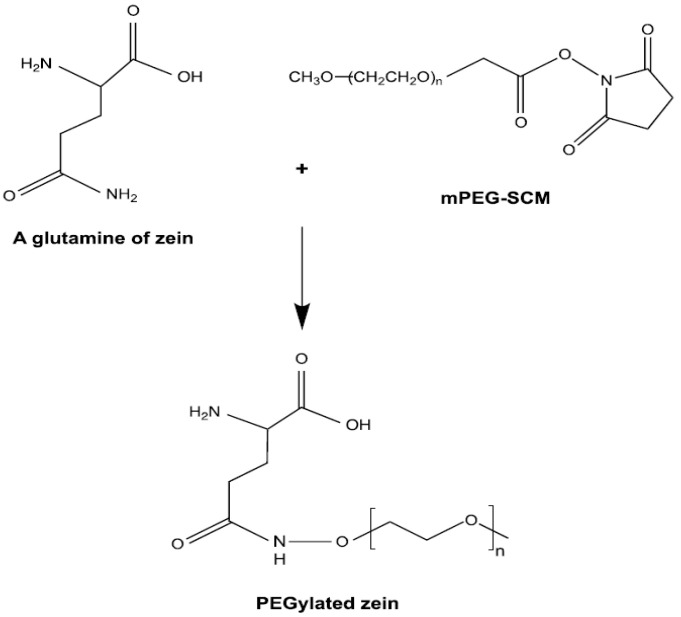
Schematic reaction of the PEGylation of zein.

PEGylated zein was prepared using a method adapted from Podaralla et al. [23] and Meewan et al. [16] (Figure 1). Briefly, yellow zein (0.1 g) was dissolved in 4 mL of 90% (*v*/*v*) ethanol. Methoxy PEG-succinimidyl carboxymethyl ester (mPEG-SCM) of molecular weight 5000 Da (0.05 g for mPEG5K-zein) or 10,000 Da (0.1 g for mPEG10K-zein) was separately dissolved in 1 mL of 90% (*v*/*v*) ethanol. The PEG solutions were added to the zein solution and stirred at 100 rpm for 3 h at 25 °C. The reaction was then quenched by adding 1 mL of 1 M glycine buffer, followed by 5 mL of citrate buffer (pH 7.4) to precipitate the PEGylated zein. The resulting mixtures were dialyzed against 2 L of ultrapure Milli-Q water (15.0 MΩ·cm) using dialysis tubing with molecular weight cut-offs of 7000 Da (for mPEG5K-zein) and 10,000 Da (for mPEG10K-zein). Dialysis was carried out for 48 h under continuous stirring (120 rpm) at 25 °C, with the water replaced three times to ensure the removal of free PEG and residual ethanol. The final products were lyophilized using a Christ Epsilon 2-4 LSC^®^ freeze-dryer (Osterode am Harz, Germany) and stored at −20 °C until use.

#### 2.2.2. Preparation of PEGylated Zein-Based Micelles Loading Docetaxel

PEGylated zein micelles loading docetaxel (mPEG-zein-DTX) were prepared by dissolving 0.3 mg of docetaxel and 50 mg of PEGylated zein (mPEG5K-zein or mPEG10K-zein) in 10 mL of 90% (*v*/*v*) ethanol. The solution was stirred overnight at 37 °C to facilitate the incorporation of docetaxel into the micellar structures. Free docetaxel and residual ethanol were removed by dialysis using SnakeSkin^®^ dialysis tubing with molecular weight cut-offs of 7000 Da (for mPEG5K-zein) and 10,000 Da (for mPEG10K-zein). Dialysis was performed against 2 L of ultrapure Milli-Q water under continuous stirring at 25 °C for 48 h, with water replaced three times during the process. The final formulations were lyophilized using a Christ Epsilon 2-4 LSC^®^ freeze-dryer (Osterode am Harz, Germany) and kept at −20 °C until further use.

#### 2.2.3. Conjugation of Transferrin to PEGylated Zein-Based Micelles Loading Docetaxel

Transferrin (Tf) was conjugated to docetaxel-loaded PEGylated zein micelles via a thiol–maleimide “click” reaction, as described by Hermanson [33], with modifications from Laskar et al. [34]. Briefly, thiolated transferrin was prepared by dissolving 10 mg of transferrin in 1 mL of 50 mM sodium phosphate buffer containing 0.15 M sodium chloride (pH 8.0). A 10-fold molar excess of Traut’s reagent (2-iminothiolane hydrochloride; 85 µL of a 2 mg/mL solution in deionized water) was added to the transferrin solution and stirred at 350 rpm for 2 h at 25 °C. The thiolated transferrin was then purified to remove unreacted Traut’s reagent by centrifugation at 4200× *g* (3140 rpm) for 15 min at 20 °C using Vivaspin^®^ 6 centrifugal filter units with a 5000 Da molecular weight cut-off (Thermo Scientific Heraeus Megafuge^®^ 16R, Waltham, MA, USA). Immediately following purification, 1 mL of PEGylated zein micelle solution (prepared by dissolving 35 mg of freeze-dried PEGylated zein in 1 mL of 50 mM sodium phosphate buffer with 0.15 M sodium chloride, pH 8.0) was added to the thiolated transferrin. The mixture was stirred continuously at 350 rpm for 1 h at 25 °C to facilitate conjugation. Unconjugated transferrin was removed using Vivaspin^®^ 6 centrifugal filter units with a 100,000 Da molecular weight cut-off, by centrifugation at 4200× *g* (3140 rpm) for 10 min at 20 °C. The final transferrin-conjugated micelles were collected and adjusted to a final volume of 1 mL using 50 mM sodium phosphate buffer containing 0.15 M sodium chloride (pH 8.0) and stored at 4 °C until further use.

### 2.3. Characterization of Tf-Bearing, PEGylated Zein-Based Micelles

To characterize the core–shell structure of the micelles, Tf-bearing, PEGylated zein-based micelles (5 mg/mL in deuterium oxide (D_2_O) and deuterated dimethyl sulfoxide (DMSO-d_6_)) were analyzed by proton nuclear magnetic resonance (^1^H NMR). ^1^H NMR spectra were acquired at 500 MHz using a Bruker Avance^®^ III HD500 NMR spectrometer (Billerica, MA, USA). The core–shell structure was confirmed by comparing spectral differences between DMSO-d_6_ (a good solvent for both core and shell) and D_2_O (a selective solvent for the hydrophilic shell), indicating the successful formation of micelles with a hydrophobic core and hydrophilic PEG-based shell.

#### 2.3.1. Determination of Critical Micelle Concentration (CMC) of Tf-Bearing, PEGylated Zein-Based Micelles

The self-assembly behavior of transferrin-bearing PEGylated zein (Tf-mPEG-zein) into micelles was assessed using the steady-state fluorescence technique with Nile red as a hydrophobic fluorescent probe. Briefly, 32 µL of Nile red solution (1 mg/mL in methanol) was added to 7 mL plastic vials. Methanol was allowed to completely evaporate over 5 h at room temperature, leaving a thin film of Nile red. Subsequently, 1 mL of Tf-mPEG5K-zein or Tf-mPEG10K-zein solutions (prepared at concentrations ranging from 0.002 to 1 mg/mL in 5% *w*/*v* glucose solution) was added to the vials, yielding a final Nile red concentration of 100 µM. Samples were vortexed for 1–2 min and incubated overnight at 25 °C in the dark. On the following day, fluorescence measurements were performed using a Varian Cary Eclipse^®^ spectrofluorometer (Agilent Technologies, Santa Clara, CA, USA) with an excitation wavelength of 550 nm and emission wavelengths from 570 to 800 nm. Excitation and emission slit widths were set at 5 nm and 20 nm, respectively. The ratio of Nile red emission intensity at the wavelength of maximum emission (λ_max_) in the presence of transferrin-bearing, PEGylated zein micelles (I) over the fluorescence in their absence (I_0_), in glucose solution (5% *w*/*v*) was plotted as a function of transferrin-bearing, PEGylated zein micelle concentration. The critical micelle concentration was determined from the inflection point of the plot of relative fluorescence intensities of Nile red at various micelle concentrations.

#### 2.3.2. Morphological Characterization by Transmission Electron Microscopy

The morphology of conjugated and non-conjugated PEGylated zein micelles (0.4 mg/mL in distilled water) was examined using transmission electron microscopy (TEM). Formvar/carbon-coated copper grids (400 mesh) were glow-discharged to render the surface hydrophilic. A 3–5 μL drop of each micelle sample was placed onto the prepared grids and allowed to air-dry overnight at room temperature. The dried samples were then imaged using a Jeol JEM-1200EX^®^ transmission electron microscope (Jeol, Tokyo, Japan) operating at an accelerating voltage of 80 kV.

#### 2.3.3. Determination of Size and Zeta Potential of the Tf-Bearing PEGylated Zein-Based Micelles

The hydrodynamic size and zeta potential of Tf-bearing and control PEGylated zein micelles (mPEG5K-zein and mPEG10K-zein) loaded with docetaxel were measured using dynamic light scattering (DLS) and laser Doppler electrophoresis, respectively. Measurements were performed with a Zetasizer Nano-ZS^®^ (Malvern Instruments Ltd., Malvern, UK) at 25 °C, with a backscattering detection angle of 173°. All samples were prepared at a concentration of 0.2 mg/mL in 5% (*w*/*v*) glucose solution, with a final volume of 1 mL. Prior to analysis, targeted micelles were lyophilized using a Christ Epsilon 2-4 LSC^®^ freeze-dryer (Osterode am Harz, Germany) and reconstituted as described before. Each measurement was conducted in quadruplicate to ensure reproducibility.

#### 2.3.4. Determination of Docetaxel Encapsulation Efficiency and Drug Loading in Tf-Bearing PEGylated Zein-Based Micelles

To determine the encapsulation efficiency of docetaxel, 1 mg of docetaxel-loaded PEGylated zein micelles was dispersed in 1 mL of ultrapure water and centrifuged at 5000× *g* for 14 min using an IEC Micromax^®^ centrifuge (Thermo Life Sciences, Waltham, MA, USA). The supernatant was discarded, and the resulting pellet was dissolved in 1 mL of methanol. An aliquot of the methanolic solution was further diluted with methanol to fall within the calibration range. The fluorescence intensity of fluorescein-labelled docetaxel was measured using a Varian Cary Eclipse^®^ fluorescence spectrophotometer (Agilent Technologies, Santa Clara, CA, USA) at an excitation wavelength of 495 nm and emission wavelength of 525 nm. Docetaxel concentration was determined by interpolating the fluorescence intensity against a standard calibration curve of fluorescein-labelled docetaxel (0.2–3 μg/mL). Encapsulation efficiency (%EE) was calculated using the following equation, with measurements performed in quadruplicate (n = 4):

%EE = (amount of docetaxel encapsulated/total amount of docetaxel added) × 100

For transferrin-conjugated micelles, 10 μL of micellar dispersion was diluted with 990 μL of 50 mM sodium phosphate buffer containing 0.15 M sodium chloride (pH 8.0). The mixture was centrifuged at 5000× *g* for 14 min, and the supernatant was discarded. The pellet was dissolved in 1 mL of methanol and analyzed following the same procedure as for non-conjugated micelles. Docetaxel loading was expressed both as %EE and as the amount of docetaxel per mg of micelle material.

#### 2.3.5. Quantification of the Amount of Tf Conjugated to the Micelles

The amount of Tf conjugated to PEGylated zein micelles was quantified using the Lowry protein assay, following the original method by Lowry et al. [35] with modifications based on a previously established protocol [36]. Briefly, Solution A was prepared by adding 1 mL of 2% (*w*/*v*) sodium potassium tartrate and 1 mL of 1% (*w*/*v*) cupric sulphate (both in ultrapure Milli-Q water, 15.0 MΩ·cm) dropwise under continuous stirring into 50 mL of 2% (*w*/*v*) anhydrous sodium carbonate in 0.1 M NaOH to prevent precipitation. A series of standard protein solutions was prepared using bovine serum albumin (BSA) in 50 mM sodium phosphate buffer containing 0.15 M sodium chloride (pH 8.0), at concentrations ranging from 5 to 500 μg/mL. For each assay, 100 μL of diluted micelle samples (prepared by mixing 5 μL of Tf-bearing or control PEGylated zein micelles with 95 μL of the same phosphate buffer) was mixed with 1 mL of Solution A and incubated at 25 °C in the dark for 10 min. Then, 100 μL of 1N Folin–Ciocalteu’s phenol reagent was added to each sample with immediate vortexing, followed by a 30 min incubation at 25 °C, protected from light. Absorbance was measured at 750 nm using a Varian Cary^®^ 50 UV-Vis spectrophotometer (Agilent Technologies, Santa Clara, CA, USA). Blank micelles were used as a reference. All measurements were performed in quadruplicate (n = 4).

### 2.4. In Vitro Studies

#### 2.4.1. Cellular Uptake

The cellular uptake of fluorescein-labelled docetaxel entrapped in transferrin-bearing and control PEGylated zein micelles, as well as in solution, was qualitatively evaluated using confocal microscopy. PC3-Luc, DU145, and LNCaP prostate cancer cells were seeded on sterile coverslips placed in 6-well plates at a density of 2 × 10^5^ cells per well and incubated for 24 h at 37 °C in a humidified atmosphere containing 5% CO_2_. Following incubation, the medium was removed, and cells were treated with fluorescein-labelled docetaxel (20 μg/well) either in solution or entrapped in the various micellar formulations (Tf-bearing and control PEGylated zein micelles of 5 K and 10 K molecular weights). Incubation with formulations was carried out for 4 h at 37 °C under the same humidified CO_2_ atmosphere. Untreated cells served as negative controls. After treatment, cells were washed three times with 3 mL of phosphate-buffered saline (PBS) and fixed with 2 mL of methanol for 10 min at 20 °C. Methanol was then removed, and the cells were left to air dry for an additional 10 min at 20 °C. Coverslips were mounted onto glass microscope slides using Vectashield^®^ mounting medium containing DAPI and allowed to stain for 2 h. Fluorescence imaging was performed using a Leica TCS SP5^®^ confocal microscope (Leica Microsystems, Wetzlar, Germany). DAPI-stained nuclei were excited with a 405 nm laser (emission bandwidth: 415–491 nm), while fluorescein-labelled docetaxel was excited with a 494 nm laser (emission bandwidth: 510–530 nm).

The cellular uptake of fluorescein-labelled docetaxel carried by transferrin-bearing and control PEGylated zein micelles, as well as in solution, was also quantitatively assessed by flow cytometry. Uptake was determined by measuring the fluorescence intensity of fluorescein-labelled docetaxel internalized by the cells. Untreated cells served as a negative control. PC3-Luc, DU145, and LNCaP prostate cancer cells were seeded into 6-well plates at a density of 1 × 10^6^ cells/well and incubated at 37 °C in a humidified 5% CO_2_ atmosphere for 24 h. After incubation, the culture medium was removed and replaced with fresh medium containing fluorescein-labelled docetaxel at a concentration of 20 μg/mL, either free or encapsulated in Tf-bearing or control PEGylated zein micelles. Cells were incubated for an additional 4 h under the same conditions. Following treatment, cells were washed three times with 2 mL of cold PBS, detached using 250 µL of TrypLE^®^ Express, and incubated for 5–10 min at 37 °C. The trypsinization reaction was stopped by adding 500 µL of 1% fetal bovine serum (FBS) in PBS. The cell suspensions were then analyzed using an Attune^®^ NxT Acoustic Focusing Cytometer (Thermo Fisher Scientific, Waltham, MA, USA) equipped with the Attune^®^ NxT Auto Sampler. A total of 30,000 gated events were recorded per sample.

#### 2.4.2. Mechanisms of Cellular Uptake

To elucidate the mechanisms involved in the endocytosis-mediated uptake of fluorescein-labelled docetaxel, PC3-Luc prostate cancer cells were treated with specific endocytic inhibitors. Cells were seeded into 6-well plates at a density of 1 × 10^6^ cells/well and incubated for 24 h at 37 °C in a humidified 5% CO_2_ atmosphere. Following incubation, the culture medium was removed, and cells were pre-treated with one of the following endocytosis inhibitors for 30 min at 37 °C: transferrin (50 µM; a competitive ligand for transferrin receptors), chlorpromazine (20 µg/mL; clathrin-mediated endocytosis inhibitor), filipin (5 µg/mL; caveolae-mediated endocytosis inhibitor), colchicine (40 µg/mL; microtubule polymerization inhibitor), or poly-L-lysine (40 µg/mL; a general inhibitor of endocytic uptake via charge neutralization). After pre-treatment, the inhibitors were replaced with fresh medium containing both fluorescein-labelled docetaxel (20 µg/well) entrapped in transferrin-bearing PEGylated zein micelles and the corresponding inhibitor at the same concentration. Cells were incubated for an additional 4 h at 37 °C. Following treatment, cells were processed for flow cytometry and confocal microscopy, as described above, to assess changes in cellular uptake.

#### 2.4.3. Anti-Proliferative Efficacy

The anti-proliferative efficacy of docetaxel entrapped in targeted and control PEGylated zein micelles or in solution was assessed using an MTT assay in PC3-Luc, DU145, and LNCaP prostate cancer cells. Cells were seeded into 96-well plates at a density of 2000 cells/well and incubated for 72 h at 37 °C in a humidified atmosphere containing 5% CO_2_. Following incubation, the medium was replaced with 200 µL of fresh medium containing various concentrations of docetaxel (ranging from 0.000001 to 60 µg/mL), either free or encapsulated in Tf-bearing or control PEGylated zein micelles. The cells were further incubated for 72 h under the same conditions. After treatment, 50 µL of MTT solution (0.5% *w*/*v* in PBS) was added to each well, and the plates were incubated for an additional 4 h to allow formation of the formazan product. The MTT solution was then removed, and 200 µL of dimethyl sulfoxide (DMSO) was added to each well to solubilize the purple formazan crystals. The absorbance was measured at 570 nm using a Multiskan Ascent^®^ microplate reader (Thermo Labsystems, Beverly, MA, USA). Percentage cell viability was calculated relative to untreated control cells. Three independent experiments were performed for each formulation, with five replicate wells (n = 5) per concentration. Triton X-100 (0.1% *w*/*v* in PBS) served as a positive control, and fresh medium was used as a negative control. Dose–response curves were constructed by plotting percentage cell viability against docetaxel concentration to evaluate the anti-proliferative efficacy of each formulation.

### 2.5. Statistical Analysis

Results were expressed as the mean ± standard error of the mean (S.E.M.) using Origin 2021 software (OriginLab Corporation, Northampton, MA, USA). Statistical analysis was performed using one-way analysis of variance (ANOVA), followed by Tukey’s multiple comparison post-test to evaluate differences between treatment groups. Analyses were conducted using Minitab^®^ software (version 22.0, Minitab LLC, State College, PA, USA). Differences were considered statistically significant at *p* < 0.05.

## 3. Results

### 3.1. Characterization of Tf-Bearing, PEGylated Zein-Based Micelles

#### 3.1.1. ^1^H-NMR Analysis

Transferrin was successfully conjugated to PEGylated zein micelles with various PEG chain lengths (5 K and 10 K Da), as confirmed by proton nuclear magnetic resonance. Proton NMR spectra of transferrin-bearing and control PEGylated zein micelles were recorded in both deuterated dimethyl sulfoxide (DMSO-d_6_) and deuterium oxide (D_2_O).

**Figure 2:**
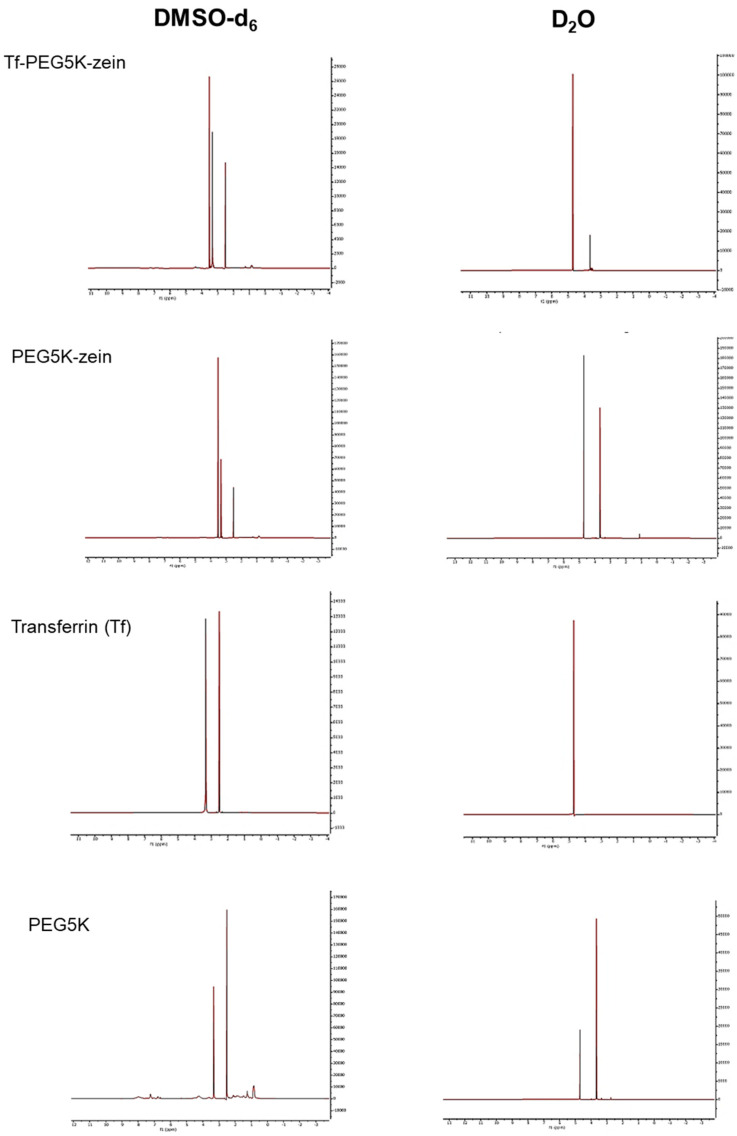
^1^H-NMR spectra of transferrin-bearing PEG5K-zein, PEG5K-zein, PEG5K, and Tf in DMSO-d_6_ (**left**) and D_2_O (**right**).

**Figure 3:**
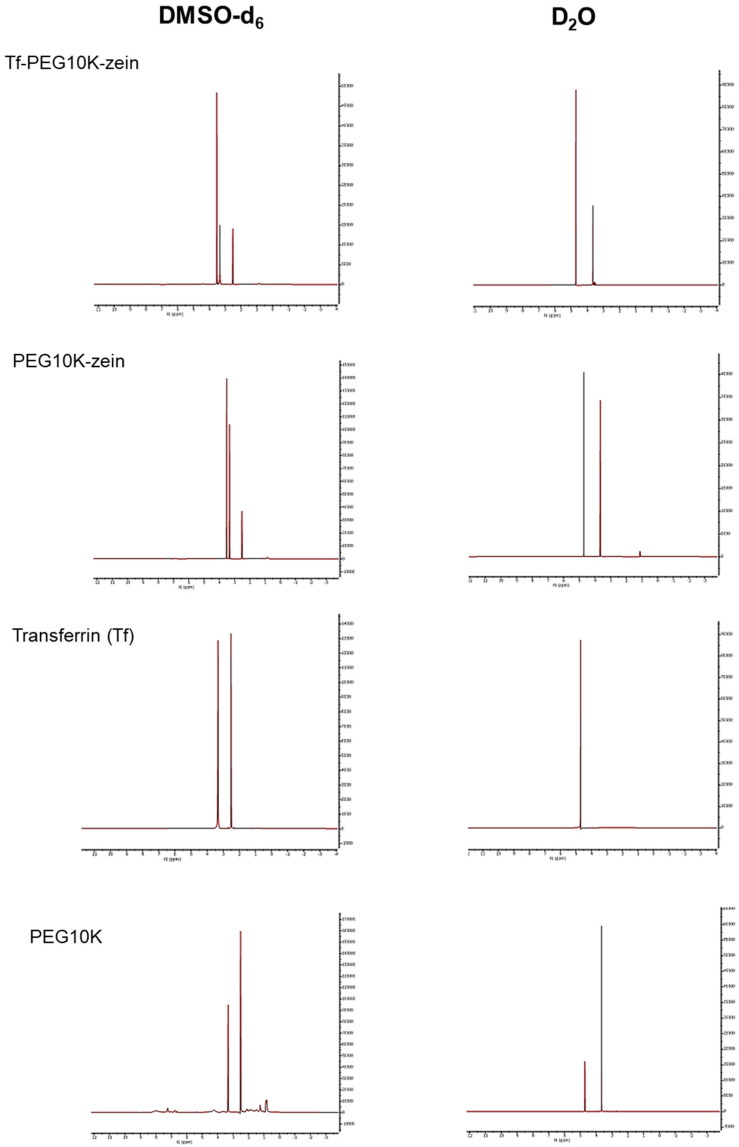
^1^H-NMR spectra of transferrin-bearing PEG10K-zein, PEG10K-zein, PEG10K, and Tf in DMSO-d_6_ (**left**) and D_2_O (**right**).

In DMSO-d_6_, where micelle formation does not occur, both the hydrophilic PEG and hydrophobic zein components were dissolved, and their characteristic proton signals were observed. Specifically, the ethylene protons of PEG appeared at δ 3.50 ppm, while the amide protons of zein were detected at δ 3.31 ppm (Figure 2 and Figure 3).

In contrast, when micelles were analyzed in D_2_O, a solvent in which only the hydrophilic shell dissolves, only the PEG signals were observed. The ethylene proton peak of PEG was shifted to δ 3.65 ppm, while the signal corresponding to the zein core was absent, indicating its sequestration in the micelle interior. This solvent-dependent shift and disappearance of the zein signal confirmed the formation of a core–shell architecture, with PEG forming the hydrophilic outer shell and zein constituting the hydrophobic core (Figure 2 and Figure 3).

In addition, the successful conjugation of transferrin to the micelles was confirmed by the appearance of a new proton signal at δ 1.3–1.5 ppm in the spectra of transferrin-conjugated micelles. This peak, which was absent in control (non-conjugated) micelles, corresponds to transferrin and matches its characteristic signal in DMSO-d_6_.

The characteristic proton resonances were as follows: PEG ethylene protons at δ 3.50 ppm, zein amide protons at δ 3.31 ppm, transferrin at δ 1.3–1.5 ppm, and solvent peaks at δ 2.6 ppm (DMSO-d_6_) and δ 4.7 ppm (D_2_O) (Figure 2 and Figure 3).

#### 3.1.2. Determination of Critical Micelle Concentration (CMC) of Tf-Bearing, PEGylated Zein-Based Micelles

**Figure 4:**
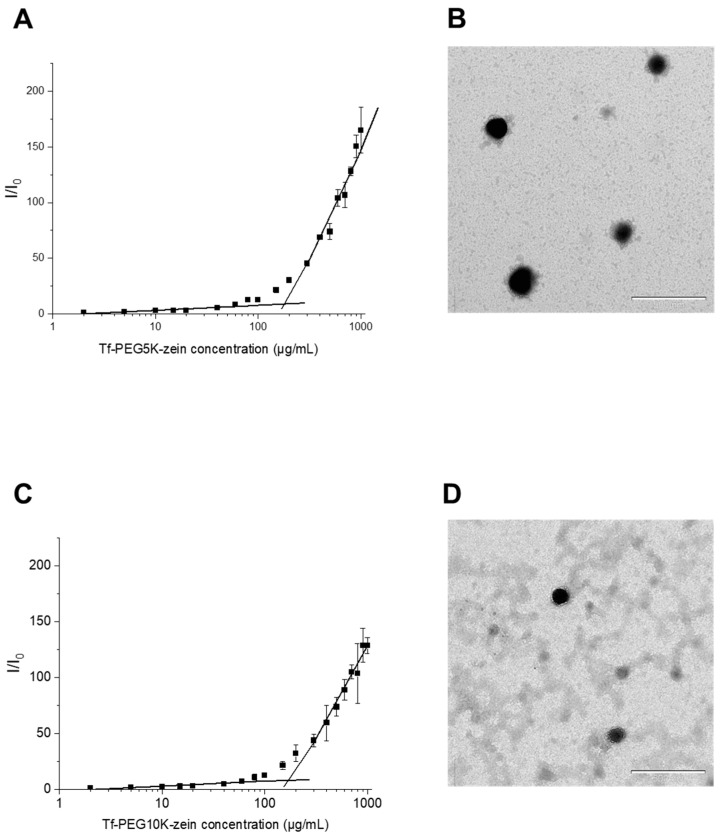
Relative fluorescence intensity (I/I_0_) of Nile Red vs. various concentrations of Tf-mPEG5K-zein (**A**) and Tf-mPEG10K-zein (**C**) (excitation and emission slits: 5 nm) (n = 3) and TEM images of Tf-mPEG5K-zein micelles (**B**) and Tf-mPEG10K-zein micelles (**D**) (scale bar: 500 nm).

The critical micelle concentrations (CMC) of transferrin-bearing, PEGylated zein micelles (Tf-mPEG5K-zein and Tf-mPEG10K-zein) were determined using Nile red as a hydrophobic fluorescent probe. Nile red exhibits minimal fluorescence in aqueous environments but becomes highly fluorescent upon partitioning into hydrophobic domains, such as the micellar core. A gradual increase in fluorescence intensity was observed as the concentration of Tf-mPEG-zein increased in 5% glucose solution, indicating the formation of micelles and the incorporation of Nile red into their hydrophobic cores. The CMC was determined from the intersection point of two linear segments on the plot of fluorescence intensity vs. Tf-mPEG-zein concentration. The CMC values were calculated to be approximately 180 ± 5 µg/mL for Tf-mPEG5K-zein micelles and 175 ± 10 µg/mL for Tf-mPEG10K-zein micelles, confirming the self-assembly capacity of both systems (Figure 4).

#### 3.1.3. Morphological Characterization by Transmission Electron Microscopy

Transmission electron microscopy revealed that both transferrin-bearing PEGylated zein micelles self-assembled into spherical structures (Figure 4). The micelle diameters were less than 500 nm, consistent with nanoscale dimensions typical of self-assembled polymeric micelles. It should be noted that TEM is performed on dried samples and typically reflects “dry” dimensions, whereas DLS measures hydrodynamic diameter in dispersion (including the solvated PEG corona and interfacial layers); therefore, DLS sizes are commonly larger than TEM sizes for soft/polymer-coated nanostructures.

#### 3.1.4. Determination of Size and Zeta Potential of the Tf-Bearing PEGylated Zein-Based Micelles

The molecular weight of PEG significantly influenced the size of both transferrin-bearing and control PEGylated zein micelles. Micelles formed with higher-molecular-weight PEG (10 K) exhibited larger sizes compared to those with lower-molecular-weight PEG (5 K). mPEG10K-zein-DTX micelles exhibited a larger diameter (186.5 ± 1.2 nm) compared to mPEG5K-zein-DTX micelles (100.8 ± 2.8 nm), consistent with a thicker hydrated PEG corona contributing to a larger hydrodynamic diameter. Transferrin conjugation led to a significant increase in the size of mPEG5K-zein micelles, whereas the size of mPEG10K-zein micelles increased only slightly upon transferrin attachment. The hydrodynamic diameters of Tf-mPEG5K-zein-DTX and Tf-mPEG10K-zein-DTX micelles were 127.7 ± 1.3 nm and 187.5 ± 1.5 nm, respectively (Table 1).

Polydispersity index (PDI) values indicated that all micelle formulations exhibited good size uniformity, with values below 0.25. The PDI values for Tf-mPEG5K-zein-DTX and Tf-mPEG10K-zein-DTX micelles were 0.19 ± 0.01 and 0.25 ± 0.01, respectively. Non-conjugated mPEG5K-zein-DTX and mPEG10K-zein-DTX micelles had PDI values of 0.20 ± 0.01 and 0.19 ± 0.00, respectively, confirming the homogeneity of the formulations (Table 1).

Zeta potential measurements showed that the prepared micelles had surface charges ranging from −24 to 30 mV, which supports their colloidal stability. Transferrin conjugation led to a reduction in zeta potential for both PEGylated micelle systems, likely due to the masking effect of the transferrin coating (Table 1).

#### 3.1.5. Determination of Docetaxel Encapsulation Efficiency and Drug Loading in Tf-Bearing PEGylated Zein-Based Micelles

The encapsulation efficiency of docetaxel in PEGylated zein micelles was determined by correlating the fluorescence intensity of each formulation with a standard calibration curve. Targeted PEGylated zein micelles (Tf-mPEG5K and Tf-mPEG10K) exhibited encapsulation efficiencies of 85.8 ± 2.8% (257.4 µg DTX) and 84.5 ± 0.6% (253.5 µg DTX), respectively. In comparison, control micelles achieved slightly higher encapsulation efficiencies of 90.5 ± 0.3% (271.5 µg DTX) and 86.7 ± 1.4% (260.1 µg DTX) for mPEG5K and mPEG10K micelles, respectively.

The amount of docetaxel loaded per mg of PEGylated zein micelles ranged narrowly across all formulations, from 5.07 to 5.43 µ/mg. Specifically, drug loading for the targeted micelles was 5.09 ± 0.13 µg/mg (5 K) and 5.07 ± 0.03 µg/mg (10 K), while the control micelles showed slightly higher values of 5.43 ± 0.01 µg/mg (5 K) and 5.20 ± 0.08 µg/mg (10 K).

#### 3.1.6. Quantification of the Amount of Tf Conjugated to the Micelles

The amount of transferrin conjugated to the PEGylated zein micelles was quantified by UV absorbance using the Lowry assay. Sample absorbance values were correlated with a standard calibration curve generated using bovine serum albumin (BSA), described by the regression equation y = 0.00138x − 0.0055, with a high coefficient of determination (R^2^ = 0.99859).

Transferrin was successfully conjugated to the micelles at substantial levels. For PEGylated zein micelles with PEG chain lengths of 5 K and 10 K, the conjugation efficiencies were 83.6 ± 0.7% and 56.0 ± 0.3%, respectively. These correspond to approximately 8.36 mg and 5.6 mg of transferrin bound per formulation, respectively. Normalized to the micelle mass used for the conjugation step (35 mg), this corresponds to 239 µg Tf/mg micelles (~3.0 nmol Tf/mg) for Tf-PEG5K-zein-DTX and 160 µg Tf/mg micelles (~2.0 nmol Tf/mg) for Tf-PEG10K-zein-DTX (assuming Tf MW ≈ 80 kDa).

### 3.2. In Vitro Studies

#### 3.2.1. Cellular Uptake

The cellular uptake of fluorescein-labelled docetaxel, either in free solution or encapsulated within transferrin-bearing and control PEGylated zein micelles (5 K and 10 K), was qualitatively assessed by confocal microscopy and quantitatively assessed by flow cytometry in PC-3-Luc, DU145, and LNCaP prostate cancer cells.

Docetaxel was predominantly localized in the cytoplasm of prostate cancer cells following treatment with all PEGylated zein micelle formulations, confirming successful intracellular delivery of the drug.

**Figure 5:**
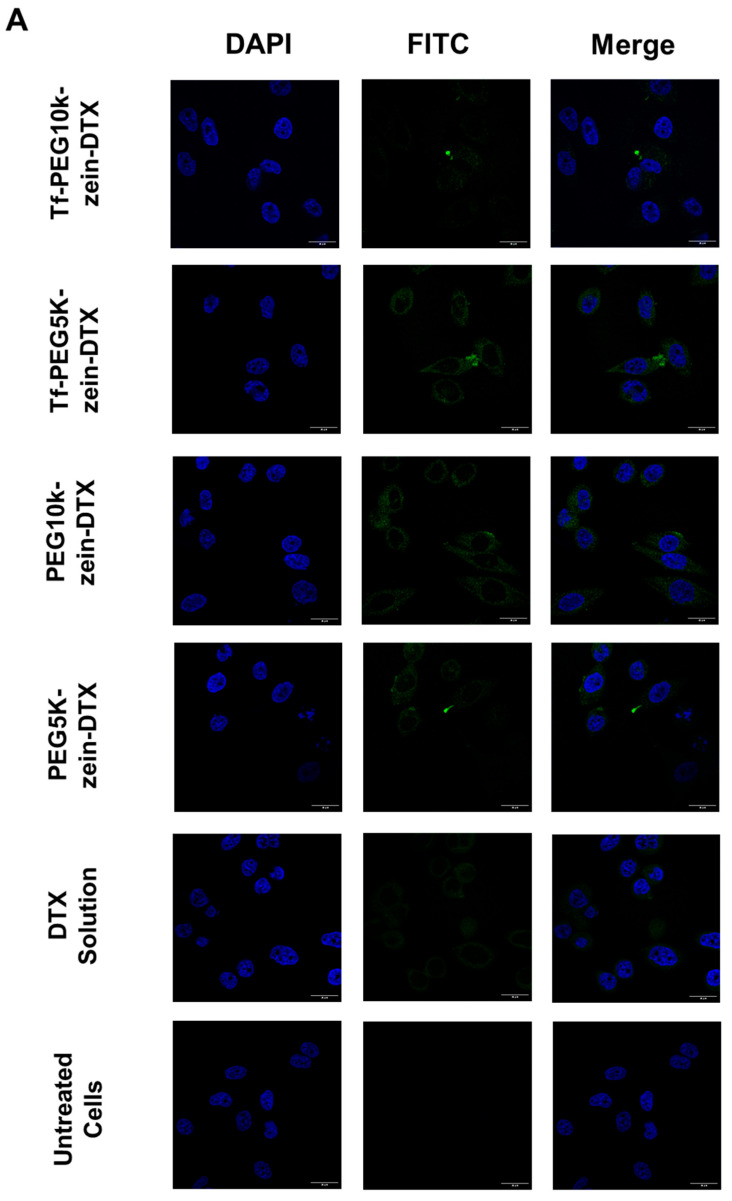

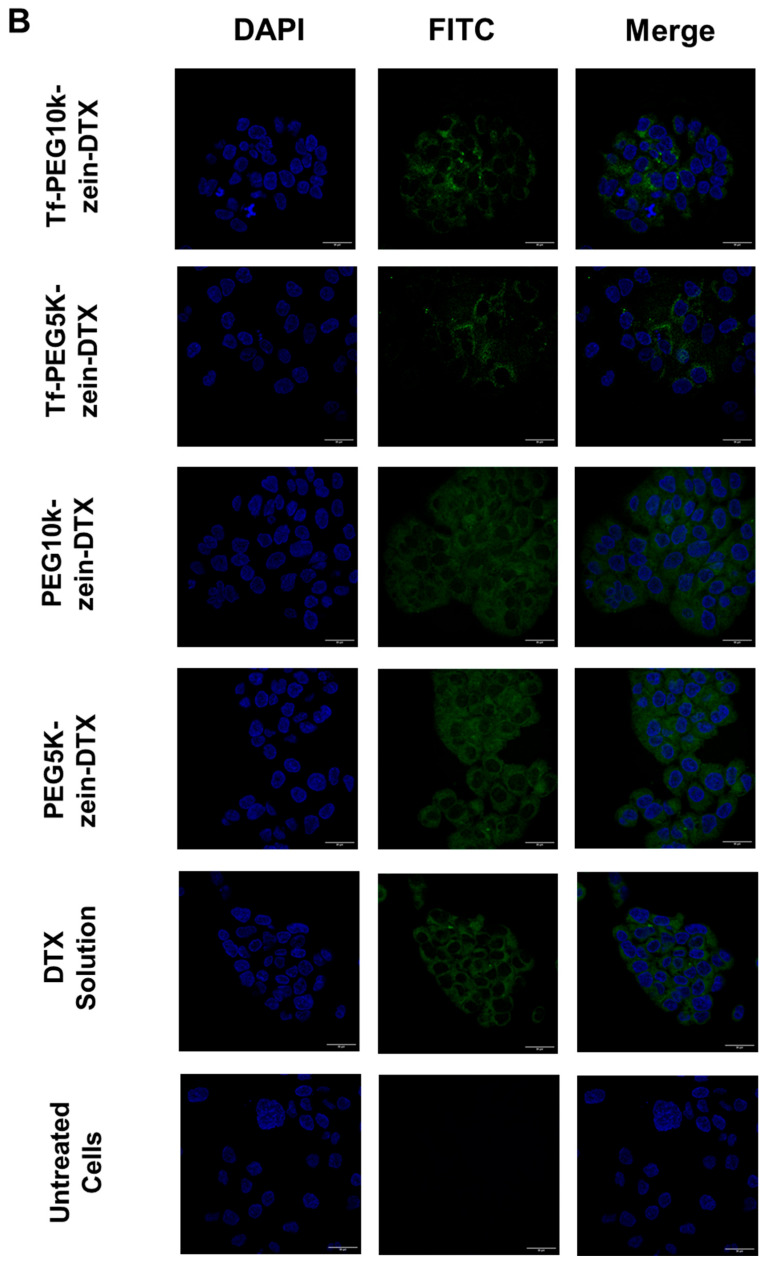

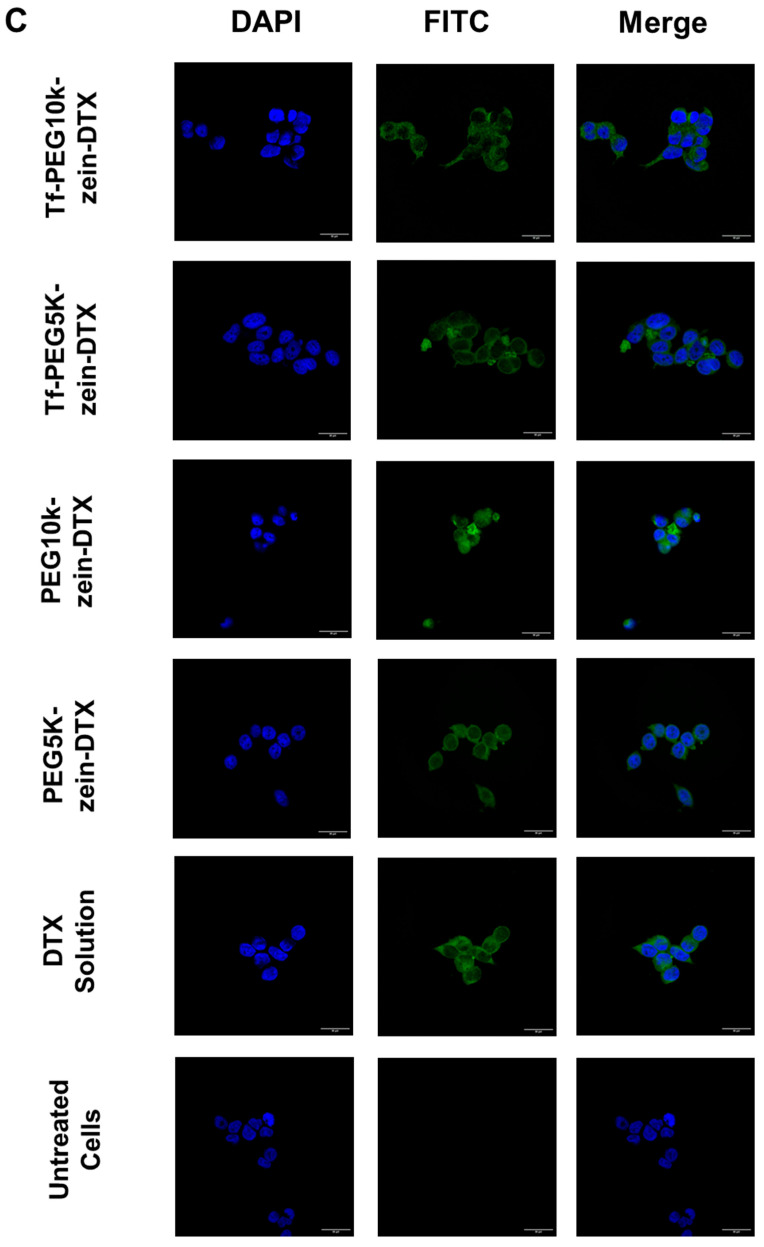
Confocal microscopy images of the cellular uptake of fluorescein-labelled docetaxel (20 μg/well) entrapped in transferrin-bearing and control m-PEG10K-zein and m-PEG5K-zein as well as in solution, after incubation for 4 h with PC-3-Luc (**A**), DU145 (**B**) and LNCaP cells (**C**). Blue: nuclei stained with DAPI (excitation: 405 nm laser line, emission bandwidth: 415–491 nm), green: fluorescein-labelled docetaxel (excitation: 494 nm laser line, bandwidth: 510–530 nm) (Bar size: 30 μm).

In PC-3-Luc cells, transferrin-bearing micelles showed lower cytoplasmic uptake of docetaxel compared to control micelles, with higher uptake observed for mPEG10K-zein micelles than for mPEG5K-zein micelles. The highest uptake overall was seen with docetaxel in free solution (Figure 5A).

In DU145 cells, Tf-bearing micelles resulted in stronger intracellular fluorescence than control micelles, again with mPEG10K-zein micelles outperforming mPEG5K-zein micelles. As with PC-3-Luc cells, treatment with docetaxel in solution produced the highest fluorescence signal, indicating greater drug uptake (Figure 5B).

In LNCaP cells, the strongest fluorescence intensity was observed with both control mPEG10K-zein micelles and docetaxel in solution. Micelles with mPEG5K (targeted and control) exhibited the lowest uptake, although targeted micelles showed slightly higher fluorescence than their control counterparts. Targeted mPEG10K-zein micelles showed slightly improved uptake compared to 5 K formulations (Figure 5C).

Across all three cell lines, treatment with docetaxel in solution consistently led to the highest cellular accumulation of the drug.

**Figure 6:**
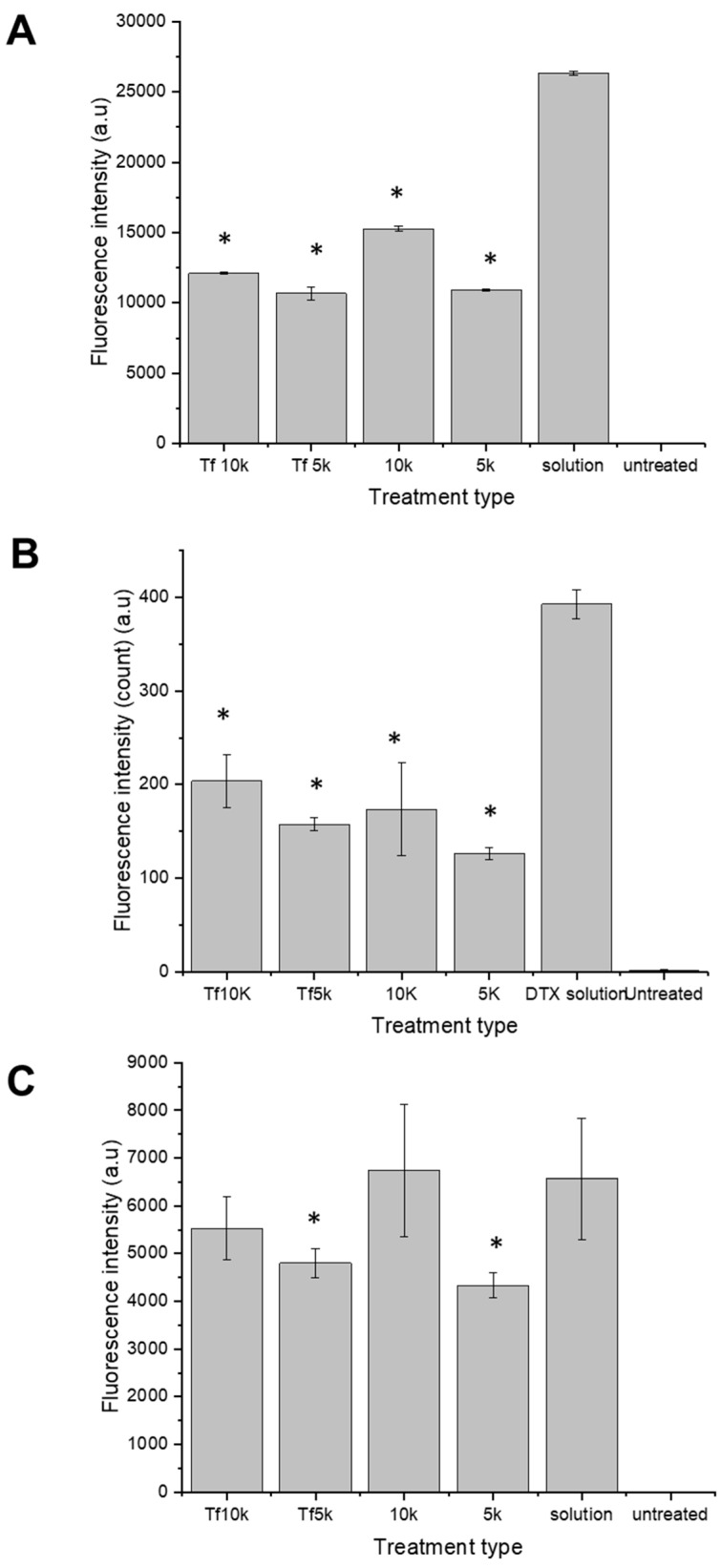
Flow cytometry quantification of the cellular uptake of fluorescein-labelled docetaxel entrapped in Tf-bearing (5 K and 10 K), control (5 K and 10 K) PEGylated zein micelles or as a solution, after incubation for 4 h with PC-3-Luc (**A**), DU145 (**B**) and LNCaP (**C**) cells (a.u.: arbitrary units) (*: *p* < 0.05 compared to the drug in solution). Results represent mean ± SEM of 3 independent experiments.

In PC-3-Luc cells, the highest uptake was observed with fluorescein-labelled docetaxel in solution, yielding a mean fluorescence intensity (MFI) of 26,327 ± 108 a.u. PEGylation significantly reduced cellular uptake compared to the free drug in solution. The MFI values were 12,111 ± 53 a.u., 10,671 ± 460 a.u., 15,267 ± 184 a.u., and 10,932 ± 64 a.u. for targeted (10 K and 5 K) and control (10 K and 5 K) PEGylated zein micelles, respectively. Control micelles, particularly the 10 K variant, showed greater uptake than transferrin-bearing counterparts. These data indicate that, at the 4 h uptake timepoint, formulations containing 10 K PEG showed higher cellular fluorescence intensity than 5 K PEG in this assay. Control mPEG10K-zein micelles delivered fluorescein-labelled docetaxel 1.2-, 1.4-, and 1.4-fold more effectively than Tf-mPEG10K-zein, Tf-mPEG5K-zein, and mPEG5K-zein micelles, respectively (Figure 6A).

In DU145 cells, the overall uptake was markedly lower than in PC-3-Luc and LNCaP cells. The highest uptake again occurred with fluorescein-labelled docetaxel in solution (MFI: 392 ± 15 a.u.). Targeted micelles showed greater uptake than control micelles. The MFI values obtained by the PEGylated micelle formulations were 204 ± 28 a.u., 157 ± 7 a.u., 173 ± 49 a.u., and 126 ± 6 a.u. for targeted (10 K and 5 K), control (10 K and 5 K) PEGylated zein micelles, respectively. Free drug in solution was the most efficacious in delivering fluorescein-labelled docetaxel into the cells by 1.9-, 2.4-, 2.2- and 3.1-fold higher compared with targeted (10 K and 5 K), control (10 K and 5 K) docetaxel-loaded PEGylated zein micelles, respectively (Figure 6B).

In LNCaP cells, the highest cellular uptakes were observed with control mPEG10K-zein micelles (MFI: 6739 ± 1393 a.u.) and the drug in solution (6568 ± 1266 a.u.). Other formulations showed lower uptake (MFI values of 5534 ± 668 a.u., 4801 ± 310 a.u., and 4334 ± 264 a.u for targeted (10 K and 5 K), and control PEGylated zein 5 K micelles, respectively) (Figure 6C).

#### 3.2.2. Mechanisms of Cellular Uptake

The mechanisms underlying the cellular uptake of fluorescein-labelled docetaxel entrapped in transferrin-bearing PEGylated zein micelles (5 K and 10 K) were investigated using specific endocytosis inhibitors and assessed both quantitatively by flow cytometry and qualitatively by confocal microscopy.

**Figure 7:**
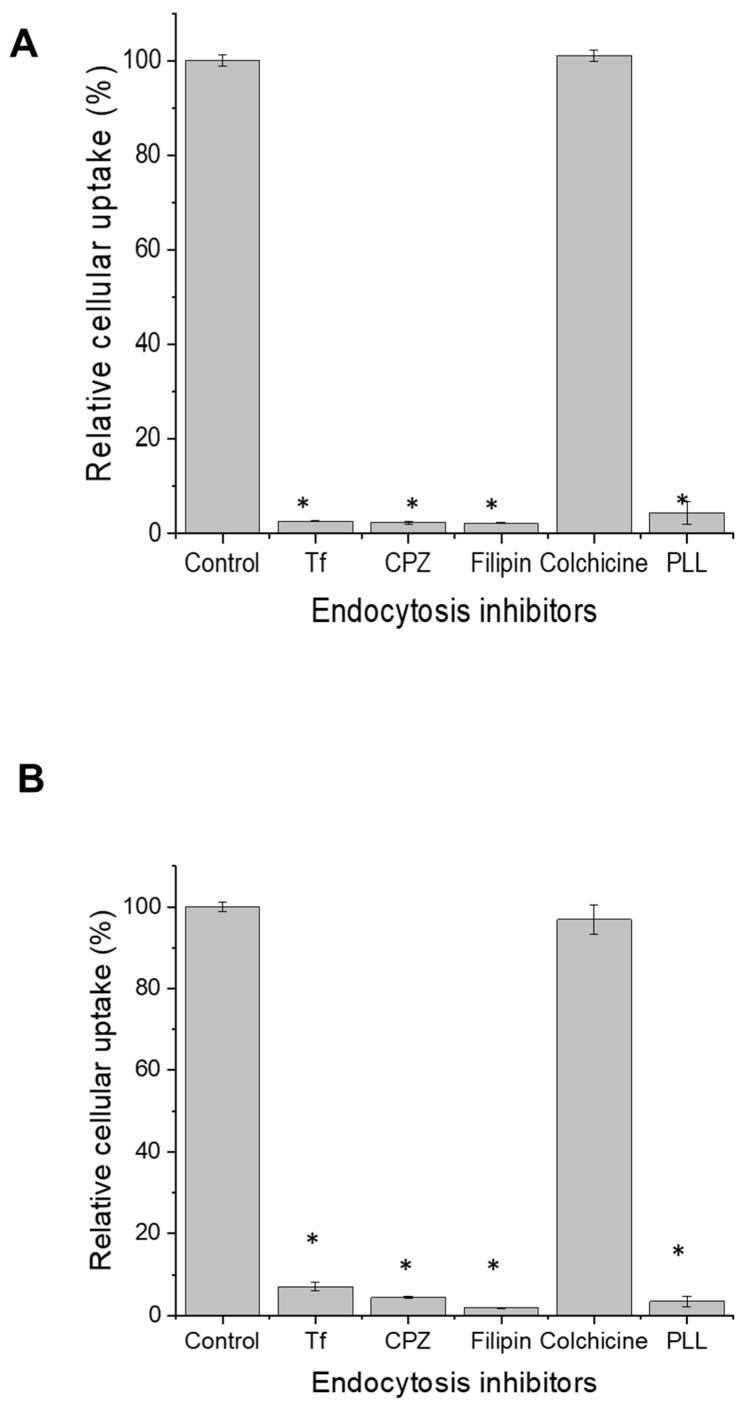
Mechanisms of cellular uptake of fluorescein-labelled docetaxel entrapped in Tf-bearing, PEGylated micelles by PC-3-Luc cells following pre-treatment with free transferrin (Tf) (50 µM), chlorpromazine (CPZ) (20 µg/mL), filipin (5 µg/mL), colchicine (40 µg/mL and poly-L-lysine (PLL) (40 µg/mL), using flow cytometry (**A**,**B**) for 5 K and 10 K PEGylated micelles, respectively (*: *p* < 0.05, compared with control with no inhibitor). Results represent mean ± SEM of 3 repeats.

In PC-3-Luc cells, pre-treatment with colchicine, a macropinocytosis inhibitor, had no effect on fluorescein-labelled docetaxel uptake for either micelle formulation, indicating that macropinocytosis was not involved in the internalization of these micelles. In contrast, other inhibitors caused significant reductions in cellular uptake (Figure 7). Filipin, a caveolae-mediated endocytosis inhibitor, nearly abolished uptake, with reductions of 97.8% and 98.2% for Tf-mPEG5K-zein and Tf-mPEG10K-zein micelles, respectively. Chlorpromazine, an inhibitor of clathrin-mediated endocytosis, caused 97.7% (5 K) and 95.6% (10 K) inhibition of fluorescein-labelled docetaxel uptake. Free transferrin, a competitor for transferrin receptors, reduced uptake by 97.4% (5 K) and 92.8% (10 K). Poly-L-lysine (PLL), which interferes with electrostatic interactions in cationic uptake pathways, inhibited uptake by 95.6% (5 K) and 96.6% (10 K). Among the inhibitors, filipin consistently caused the highest inhibition, indicating that caveolae-mediated endocytosis is the dominant pathway for micelle internalization. Significant inhibition by chlorpromazine and free transferrin also confirms involvement of clathrin-mediated and transferrin receptor-mediated endocytosis, respectively. The inhibitory effect of PLL suggests that cationic delivery mechanisms also contribute to micelle uptake.

**Figure 8:**
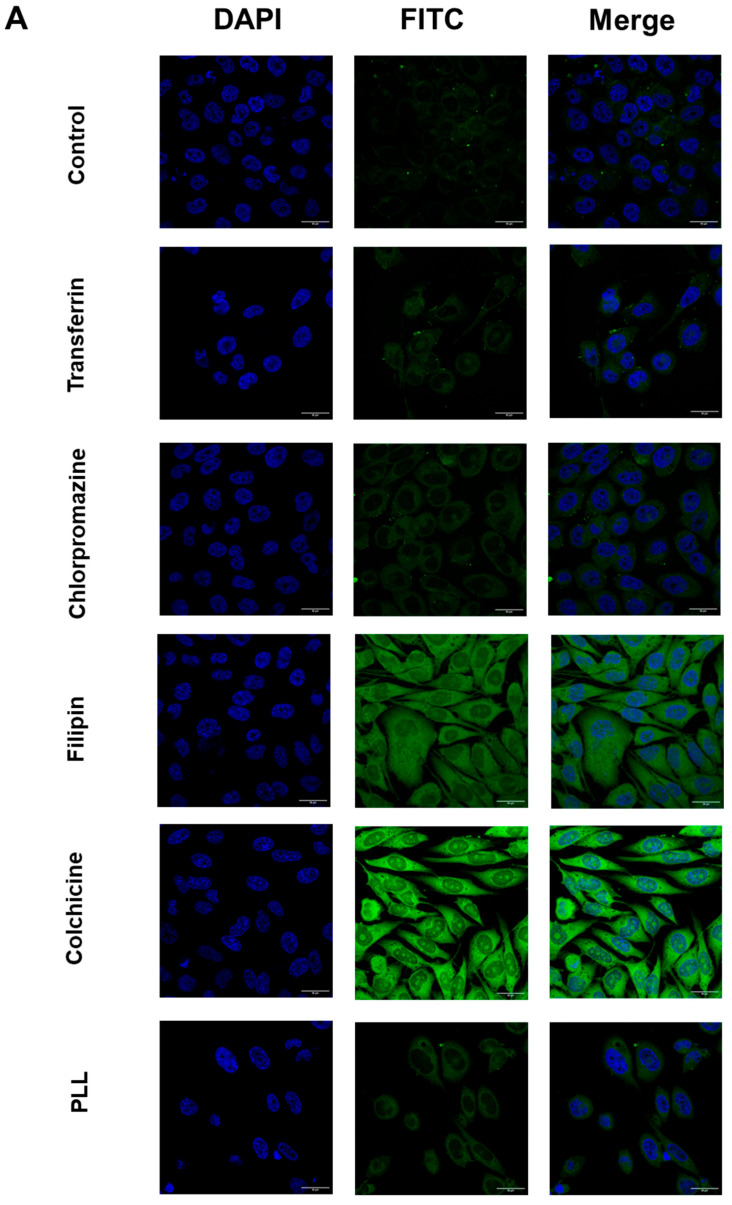

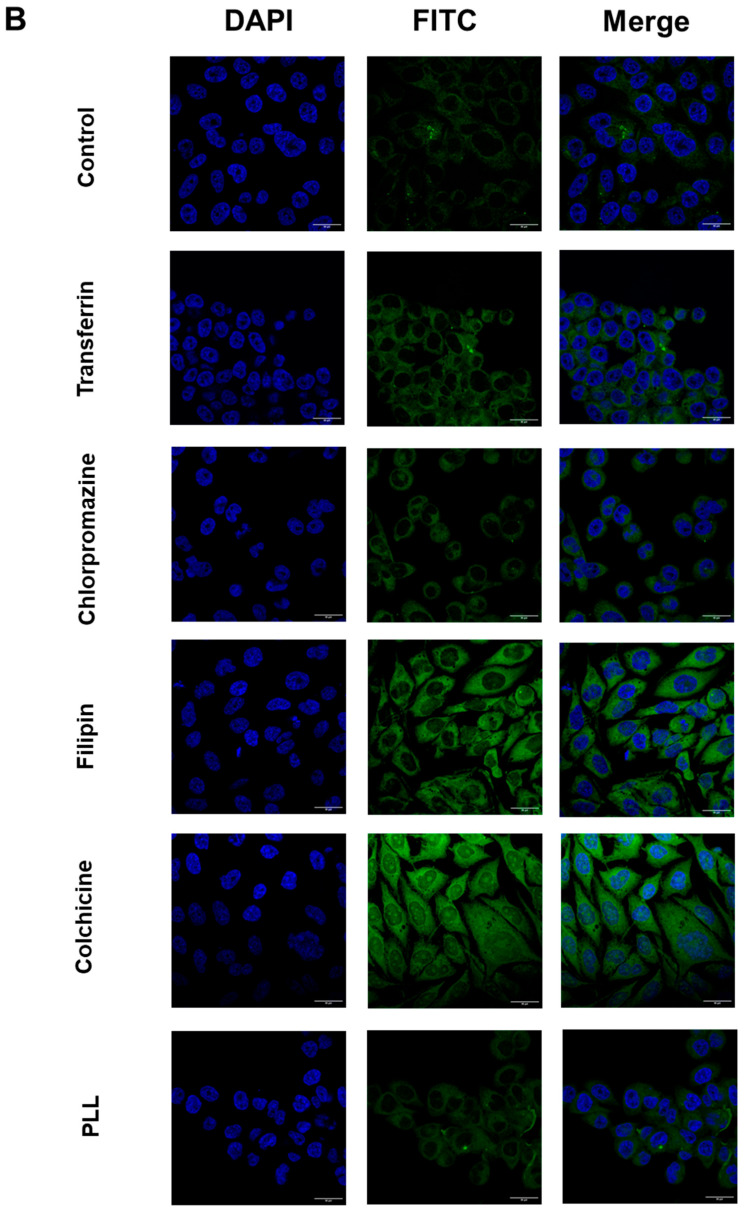
Mechanisms of cellular uptake of fluorescein-labelled docetaxel entrapped in Tf-bearing, PEGylated micelles by PC-3-Luc cells following pre-treatment with free transferrin (Tf) (50 µM), chlorpromazine (CPZ) (20 µg/mL), filipin (5 µg/mL), colchicine (40 µg/mL and poly-L-lysine (PLL) (40 µg/mL), using confocal microscopy (**A**,**B**) for 5 K and 10 K PEGylated micelles, respectively (Bar size: 30 μm).

These findings were further supported by confocal microscopy, which qualitatively confirmed the results observed in flow cytometry (Figure 8). The most substantial reduction in fluorescence was observed following filipin pre-treatment, consistent across both PEG chain lengths. In cells treated with Tf-mPEG5K-zein micelles, uptake was mainly inhibited by filipin, chlorpromazine, and free transferrin, followed by PLL. For Tf-mPEG10K-zein micelles, the uptake was most strongly inhibited by filipin, followed by PLL, chlorpromazine, and transferrin.

These results demonstrate that the internalization of transferrin-bearing PEGylated zein micelles in PC-3-Luc cells is primarily mediated by caveolae-dependent and clathrin-dependent endocytosis, along with contributions from transferrin receptor-mediated uptake and cationic delivery. Macropinocytosis did not appear to play a role, as evidenced by the lack of inhibition by colchicine.

#### 3.2.3. Anti-Proliferative Efficacy

**Figure 9:**
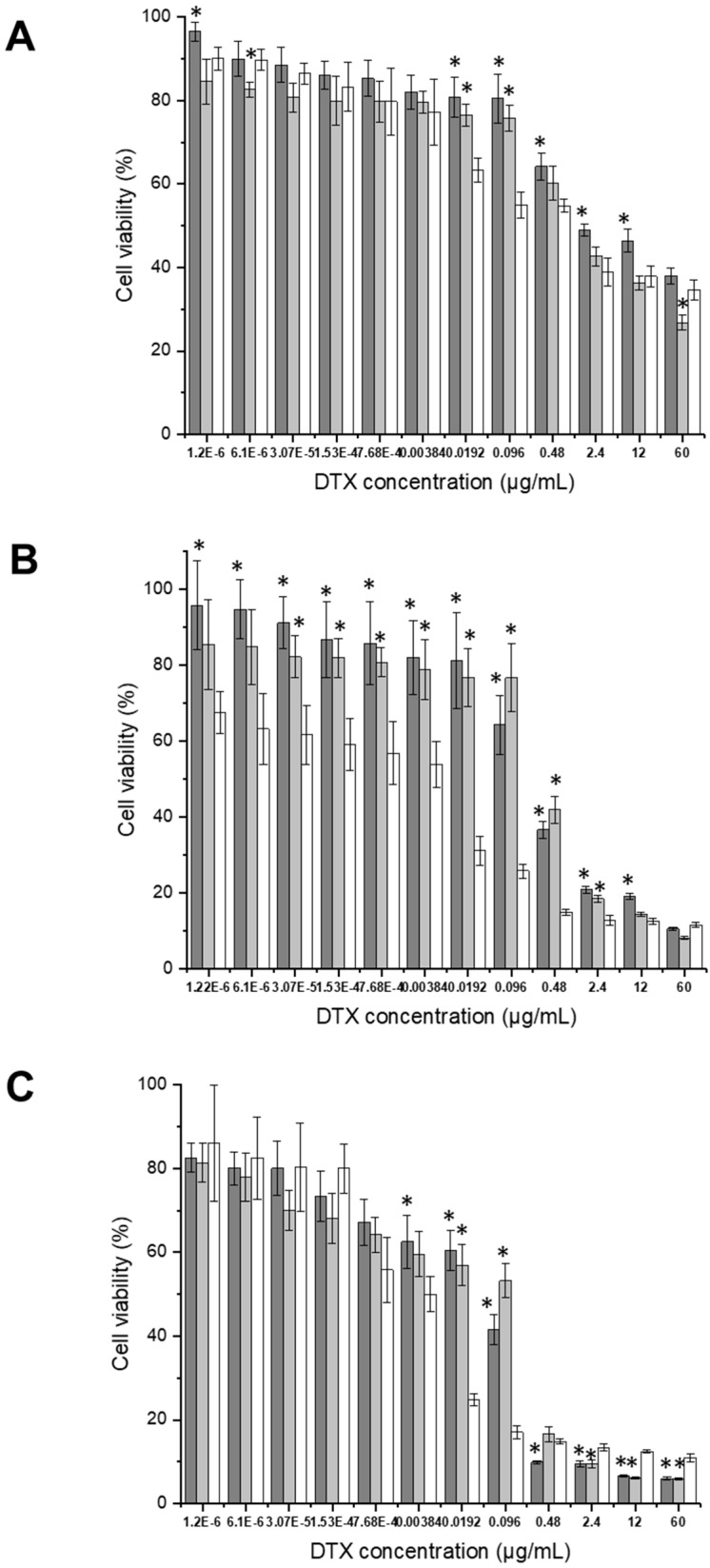
Anti-proliferative efficacy of Tf-bearing, PEGylated zein 5 K micelles entrapping docetaxel (dark grey), control PEGylated zein 5 K micelles entrapping docetaxel (grey) and docetaxel solution (white) on PC-3-Luc (**A**), DU145 (**B**), and LNCaP (**C**) prostate cancer cells (n = 15) (*: *p* < 0.05 compared to the drug in solution).

The anti-proliferative efficacy of docetaxel entrapped in PEGylated zein 5 K micelles, formulated as either transferrin-bearing or control micelles, was evaluated in comparison to free docetaxel in solution across three prostate cancer cell lines (PC-3-Luc, DU145, and LNCaP) (Figure 9). The trends were generally consistent with those observed for the 10 K PEGylated micelles, though the magnitude of the effects varied. Overall, docetaxel delivered via transferrin-bearing PEGylated 5 K zein micelles exhibited lower efficacy than that delivered via 10 K micelles, particularly in PC-3-Luc cells.

In PC-3-Luc cells, transferrin-bearing PEGylated zein 5 K micelles showed the lowest anti-proliferative effect, with the percentage cell viability of 38.0 ± 1.9% at the highest docetaxel concentration. In comparison, docetaxel in solution reduced cell viability to 34.6 ± 2.3%, while control micelles were more effective, with cell viability values of 26.7 ± 1.7% (Figure 9A).

In DU145 cells, transferrin conjugation also did not improve anti-proliferative efficacy over the free drug. Targeted micelles resulted in cell viability values of 10.6 ± 0.4% at the highest docetaxel concentrations, similar to the 11.6 ± 0.6% for docetaxel in solution and 8.1 ± 0.3% for control micelles at the same concentration (Figure 9B).

However, in LNCaP cells, transferrin-bearing PEGylated zein 5 K micelles slightly improved the anti-proliferative efficacy over docetaxel in solution, for drug concentrations higher than 0.48 µg/mL. Cell viability was reduced to 5.9 ± 0.2% at the highest concentration, compared to 10.9 ± 0.8% for the drug in solution. Control micelles also performed well, with viability values of 5.8 ± 0.3% (Figure 9C).

**Figure 10:**
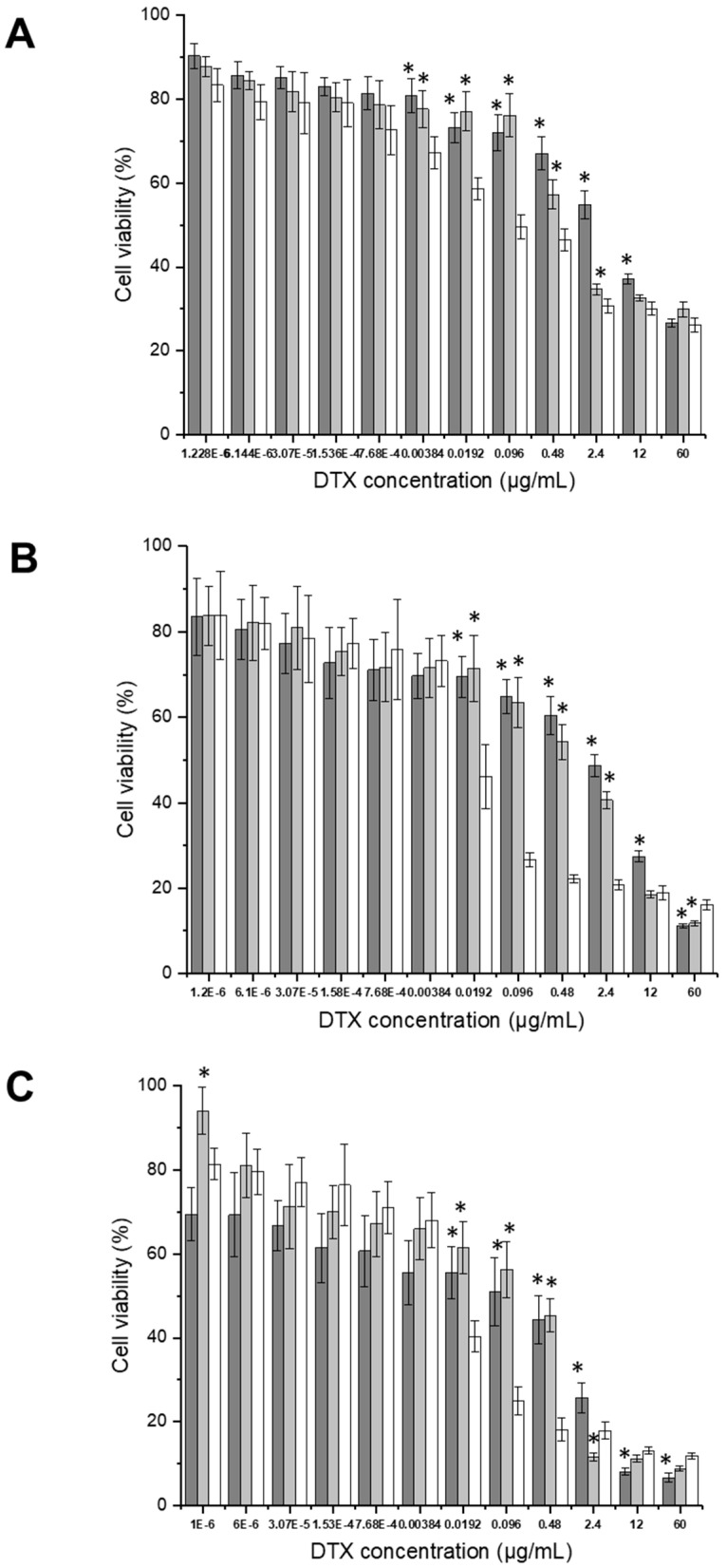
Anti-proliferative efficacy of Tf-bearing, PEGylated zein 10 K micelles entrapping docetaxel (dark grey), control PEGylated zein 10 K micelles entrapping docetaxel (grey) and docetaxel solution (white) on PC-3-Luc (**A**), DU145 (**B**), and LNCaP (**C**) prostate cancer cells (n = 15) (*: *p* < 0.05 compared to the drug in solution).

The anti-proliferative activity of docetaxel, delivered either in solution or entrapped within transferrin-bearing and control PEGylated zein micelles (10 K), was also evaluated in the 3 prostate cancer cell lines (Figure 10).

In PC-3-Luc cells, all formulations exhibited a dose-dependent reduction in cell viability. At the highest docetaxel concentration (60 µg/mL), targeted micelles slightly improved the anti-proliferative effect, reducing cell viability to 26.5 ± 0.9% (73.5% reduction), similar to that observed for the free drug (26.1 ± 1.6%, 73.9% reduction) and for control micelles (29.8 ± 1.8% viability; 70.2% reduction) (Figure 10A).

In DU145 cells, targeted micelles demonstrated a modest improvement in anti-proliferative efficacy compared to free drug at the highest concentration. At 60 µg/mL, targeted micelles reduced viability to 11.1 ± 0.3% (88.9% reduction), compared to 16.1 ± 1.1% for docetaxel in solution (83.9% reduction). The control micelles showed similar anti-proliferative efficacy at high concentration (11.7 ± 0.5% viability; 88.3% reduction) (Figure 10B).

In LNCaP cells, targeted micelles significantly enhanced the anti-proliferative efficacy at the highest drug concentrations. At 60 µg/mL, viability was reduced to 6.6 ± 1.1% (93.4% reduction) with targeted micelles, compared to 8.8 ± 0.5% (91.2% reduction) for control micelles and 11.8 ± 0.7% (88.2% reduction) for free docetaxel (Figure 10C).

Overall, docetaxel entrapped in transferrin-bearing PEGylated zein 10 K micelles demonstrated the highest anti-proliferative efficacy in LNCaP cells, followed by DU145, and then PC-3-Luc cells, with maximum reductions in cell viability of 93.4%, 88.9%, and 73.5%, respectively.

Across all prostate cancer cell lines tested (PC-3-Luc, DU145, and LNCaP), docetaxel in solution consistently exhibited the lowest IC_50_ values, indicating the highest anti-proliferative efficacy (Table 2). While encapsulation of docetaxel in PEGylated zein 5 K micelles (either targeted or control) resulted in higher IC_50_ values, transferrin conjugation significantly enhanced the anti-proliferative efficacy relative to control micelles.

In PC-3-Luc cells, the IC_50_ of docetaxel in solution was 0.04 ± 0.01 µg/mL, compared to 0.09 ± 0.04 µg/mL for transferrin-bearing micelles and 0.66 ± 0.14 µg/mL for control micelles. This reflects a 7.3-fold enhancement in efficacy with transferrin conjugation relative to control micelles.

Similarly, in DU145 cells, docetaxel in solution exhibited the lowest IC_50_ value (0.0126 ± 0.0032 µg/mL), which was 10.3-fold and 21.4-fold lower than the IC_50_ values of targeted (0.13 ± 0.05 µg/mL) and control (0.27 ± 0.09 µg/mL) micelles, respectively. Transferrin-bearing micelles demonstrated a 2-fold improvement in efficacy over control micelles.

In LNCaP cells, docetaxel in solution again showed the highest potency (IC_50_ = 0.0017 ± 0.0005 µg/mL), followed by control micelles (0.08 ± 0.03 µg/mL) and transferrin-bearing micelles (0.04 ± 0.01 µg/mL). Transferrin conjugation improved the anti-proliferative efficacy of docetaxel by 2-fold compared to the non-targeted formulation, as observed in DU145 cells.

However, in contrast, 10 K micelle formulations revealed a different trend. In all three cell lines, control micelles outperformed transferrin-bearing micelles, with lower IC_50_ values.

The IC_50_ values against the PC-3-Luc cell line were lower for docetaxel in solution, followed by 0.28 ± 0.09 µg/mL for control micelles, compared with 0.61 ± 0.31 µg/mL for targeted micelles.

In the DU145 cell line, slightly higher IC_50_ values were observed overall. The lowest IC_50_ value was 0.010 ± 0.003 µg/mL for docetaxel in solution, followed by 1.26 ± 0.28 µg/mL for the control micelles, compared with 2.67 ± 0.82 µg/mL for targeted micelles.

In LNCaP cells, docetaxel in solution exhibited the highest anti-proliferative efficacy, with the lowest IC_50_ value of 0.001 ± 0.0005 µg/mL, followed by control micelles (IC_50_: 0.04 ± 0.03 µg/mL). The targeted micelles showed the least potency in this cell line, with a significantly higher IC_50_ value of 0.71 ± 0.22 µg/mL.

These findings suggest that while transferrin conjugation improves efficacy in 5 K micelles, it does not enhance the overall performance in 10 K micelle formulations.

## 4. Discussion

This study explored the design, synthesis, and performance of transferrin (Tf)-functionalized PEGylated zein micelles as docetaxel (DTX) nanocarriers for prostate cancer, with a specific focus on how PEG chain length (5 kDa vs. 10 kDa) governs ligand presentation and downstream biological performance. PEG5K was selected as a commonly used PEG molecular weight expected to provide steric stabilization while maintaining ligand accessibility, whereas PEG10K was chosen to represent a longer PEG corona expected to increase steric shielding and therefore challenge Tf–receptor engagement [21,22]. The results demonstrate successful micelle formation, efficient docetaxel loading, and PEG-length-dependent effects of transferrin conjugation on uptake mechanisms and anti-proliferative activity in PC-3-Luc, DU145, and LNCaP cell lines. Importantly, although PEGylated zein systems and Tf targeting have been explored previously, there remains limited evidence directly comparing how PEG molecular weight modulates Tf display, receptor engagement, and biological performance within the same zein micelle platform. By comparing PEG5K versus PEG10K in otherwise closely matched formulations, we show that PEG length strongly governs Tf conjugation efficiency and functional ligand accessibility, which in turn influences uptake behavior and cytotoxicity outcomes [16,24].

NMR analyses supported the successful synthesis of PEGylated zein and subsequent Tf conjugation, consistent with the expected core–shell organization in which zein forms the hydrophobic core and PEG constitutes the hydrophilic corona [15,16]. Both PEG5K and PEG10K formulations self-assembled in aqueous medium, as reflected by low critical micelle concentrations and spherical morphologies under TEM. Any discrepancies between DLS- and TEM-derived sizes are expected because DLS reports hydrodynamic diameters in dispersion (including solvated polymer layers), whereas TEM reflects dried particle dimensions on a grid [7].

PEG chain length strongly influenced micellar size, with PEG10K micelles exhibiting larger hydrodynamic diameters than PEG5K micelles. This trend is consistent with the fact that increasing PEG molecular weight increases the thickness and steric volume of the hydrated corona, thereby increasing the measured hydrodynamic diameter [7]. Tf conjugation further increased micelle size, consistent with the addition of a surface-associated protein layer and increased interfacial hydration after ligand functionalization. The size increase after Tf attachment appeared more pronounced for the PEG5K formulation, which may reflect a larger relative contribution of the ligand layer to smaller carriers and/or differences in effective ligand presentation. All micelles maintained polydispersity indices below 0.25, indicating that PEGylation and Tf functionalization did not induce broad aggregation and preserved formulation uniformity. In addition, the micelles remained within size regimes commonly discussed as permissive for tumor extravasation in leaky vasculature models [37], noting that in vivo transport depends strongly on tumor type and physiology.

Zeta potential analysis showed that control (non-targeted) micelles were positively charged, whereas all Tf-bearing micelles carried a negative surface charge (Table 1). This shift is consistent with successful surface modification because transferrin is an acidic glycoprotein, and its surface presentation can dominate the measured electrophoretic mobility by shifting the effective shear plane and partially shielding the underlying micellar surface [26,28]. Beyond confirming conjugation, this change is relevant to nano–bio interactions because a less cationic (or anionic) surface can reduce non-specific electrostatic adsorption to cell membranes and other biological interfaces compared to a strongly cationic surface. This is pertinent here because surface charge can contribute to uptake differences between targeted and non-targeted micelles: cationic nanoparticles/micelles often exhibit enhanced cellular uptake in non-phagocytic cells via electrostatic attraction to the negatively charged plasma membrane, promoting adsorptive interactions and endocytosis [38,39,40]. In contrast, transferrin conjugation shifted the zeta potential to negative values, which may reduce non-specific electrostatic adsorption and thereby contribute to lower uptake for some Tf-bearing formulations, particularly where functional Tf–TfR engagement is limited.

High encapsulation efficiency and consistent drug loading across all formulations indicate that zein micelles are well suited for incorporating hydrophobic drugs such as docetaxel [9,11]. All micelles achieved >84% encapsulation efficiency (higher than 252 µg docetaxel), with higher encapsulation efficiency for PEG5K micelles compared with PEG10K micelles. A plausible explanation is that increasing the PEG contribution can alter core–corona packing and reduce the effective hydrophobic microenvironment available for drug partitioning into the zein core [7]. The slightly lower entrapment efficiency relative to some earlier zein-based reports may also reflect zein source/composition effects; for example, yellow zein contains additional hydrophobic components (e.g., pigments) that can influence solubility and encapsulation behavior compared with more purified forms [9,11].

Quantification of Tf conjugation efficiency revealed substantial surface modification, particularly for PEG5K micelles (83.6%) compared with PEG10K micelles (56%). This difference is consistent with steric hindrance imposed by a longer PEG corona, which can obstruct reactive groups during conjugation and can also reduce effective ligand accessibility by partially burying ligands within the PEG brush [21,25]. This PEG-length-dependent “shielding versus accessibility” trade-off provides a mechanistic basis for differences in uptake and efficacy between Tf-bearing PEG5K and PEG10K systems.

In vitro uptake studies revealed several key patterns. Across all three cell lines, free docetaxel showed the highest cellular uptake, as expected for a small molecule in 2D monolayer assays where diffusion and rapid membrane access can dominate compared with nanocarriers that require binding/internalization and intracellular release [7]. Among micellar formulations, uptake varied with PEG length, Tf conjugation, and cell line. In PC-3-Luc cells, control PEG10K micelles displayed the highest uptake among micelle formulations, while Tf-bearing micelles showed reduced internalization; this pattern is consistent with a scenario where cationic surface-driven uptake is prominent for controls, while PEG steric shielding and/or reduced functional ligand exposure limits Tf-mediated enhancement. In DU145 cells, uptake of Tf-bearing micelles was significantly higher than that of their matched controls, consistent with TfR-mediated contributions in a context where receptor availability and endocytic capacity support ligand-driven internalization. In LNCaP cells, uptake levels were more balanced between control and targeted PEG10K micelles, with a modest advantage for Tf-bearing micelles. These observations align with the broader point that receptor density and cell-specific endocytic machinery can both shape nanoparticle uptake, and that receptor expression alone may not fully predict internalization outcomes [21,27].

Mechanistic studies using pharmacological inhibitors indicated that internalization of Tf-functionalized micelles involved caveolae- and clathrin-associated pathways as well as transferrin receptor–related uptake, supported by significant inhibition with filipin, chlorpromazine, and free transferrin. Poly-L-lysine also inhibited uptake, consistent with an electrostatic component to interaction/attachment in these systems. By contrast, colchicine produced negligible effects, suggesting macropinocytosis did not play a major role under the conditions tested, consistent with prior observations for PEGylated zein micelles in another cancer model [16,17]. Collectively, these data support the view that Tf-bearing micelles exploit multiple endocytic routes, with the relative contribution of receptor-mediated versus charge-mediated interactions depending on formulation surface properties and cellular context [26,28,29,41].

Anti-proliferative efficacy further clarified the functional consequences of these uptake patterns. Docetaxel in solution consistently showed the lowest IC_50_ values across all cell lines, reflecting its rapid availability in 2D assays. When delivered via PEGylated zein micelles, potency was lower than the free drug but remained within the low µg/mL range across formulations. Among micelles, Tf-bearing PEG5K micelles markedly improved cytotoxicity relative to matched control micelles. For example, in PC-3-Luc cells, the IC_50_ for targeted PEG5K micelles was 0.09 ± 0.04 µg/mL versus 0.66 ± 0.14 µg/mL for control PEG5K micelles (7.3-fold improvement), and ~2-fold improvements were observed in DU145 and LNCaP cells. These results support the interpretation that shorter PEG chains facilitate better functional accessibility of the Tf ligand and more effective receptor-mediated contributions to uptake and activity [21,25]. In contrast, this targeting benefit was not observed for PEG10K micelles: Tf-bearing PEG10K micelles consistently exhibited higher IC_50_ values than their matched PEG10K controls across all cell lines, indicating reduced functional efficacy. This counterintuitive outcome is consistent with steric shielding by longer PEG chains, which can diminish ligand–receptor engagement and reduce the functional impact of targeting ligands [21,25]. Notably, while longer PEG chains are often used to increase steric shielding in biological media, “stealth” behavior (e.g., reduced opsonization and prolonged circulation) depends strongly on PEG surface architecture and requires dedicated protein adsorption and/or pharmacokinetic evaluation, which was outside the scope of this in vitro study [21,42].

Our findings align with the broader literature showing that transferrin functionalization can improve taxane delivery performance relative to non-targeted nanocarriers, while the magnitude and direction of benefit depend on how ligands are presented at the nanoparticle surface. Fernandes et al. reported that transferrin-conjugated liposomes enhanced docetaxel efficacy relative to non-targeted systems [30], and Zhai et al. described improved delivery efficiency and anti-proliferative activity with transferrin-modified liposomes [31]. Sahoo et al. similarly showed improved uptake and anti-proliferative efficacy of transferrin-conjugated paclitaxel nanoparticles versus unconjugated nanoparticles and drug in solution in prostate cancer models [29]. Within zein-based systems, the feasibility of combining zein with transferrin targeting has also been supported by transferrin-bearing zein hybrid lipid nanoparticles for docetaxel delivery to prostate cancer cells [24]. Against this background, the novelty of the present work lies in directly demonstrating, within the same zein micelle platform, that PEG chain length can invert the apparent benefit of Tf targeting (beneficial at PEG5K, detrimental at PEG10K), providing a clear design rule for balancing PEG-corona steric shielding with functional ligand accessibility.

Overall, these data show that transferrin-functionalized PEGylated zein micelles provide a useful platform for probing how PEG chain length controls transferrin display, receptor engagement, and cellular internalization. While free docetaxel remains the most potent in vitro in the 2D assays used here, Tf-bearing PEG5K micelles improved anti-proliferative efficacy relative to their matched non-targeted micelles, consistent with a functional contribution of transferrin when ligand masking is limited. Considering the higher transferrin conjugation efficiency for PEG5K and the more favorable IC_50_ improvements versus matched non-targeted controls, Tf-PEG5K-zein-DTX is identified as the lead targeted formulation in this study, while Tf-PEG10K-zein-DTX serves as a comparator to illustrate PEG-length/steric shielding effects. The experimental design included matched non-targeted micelles (PEG5K-zein-DTX and PEG10K-zein-DTX) as PEG-only controls, enabling separation of PEG chain-length effects from transferrin functionalization effects, and free transferrin competition provided a functional control supporting receptor involvement [26,28].

Future work could explore intermediate PEG chain lengths (e.g., PEG2K) and alternative linker/architecture strategies to improve ligand accessibility without compromising micelle integrity in biological media [21]. Given that transferrin receptor targeting is also widely investigated in other biological barriers, future studies should also consider off-target interactions and biodistribution questions in more complex models. Finally, additional comparator systems such as TfR-low cells would further strengthen selectivity assessment beyond competition assays, and inclusion of blank micelles and orthogonal structural methods could help refine interpretation of drug distribution within the micellar structure [7,27]. If development of a dried product is pursued in future, a dedicated lyophilization/redispersion optimization study (including suitable lyoprotectants) would be warranted, as freeze-drying can affect the redispersibility of self-assembled nanocarriers.

## 5. Conclusions

This study successfully developed and characterized transferrin-conjugated PEGylated zein micelles as a targeted nanocarrier system for docetaxel delivery in prostate cancer. Through physicochemical analysis and in vitro testing across three prostate cancer cell lines (PC-3-Luc, DU145, and LNCaP), the impact of PEG chain length and transferrin surface modification on micellar behavior, cellular uptake, and anti-proliferative efficacy was thoroughly investigated.

The findings demonstrated that PEG chain length plays a pivotal role in modulating micelle size, transferrin conjugation efficiency, and biological performance. Shorter PEG chains (5 K) allowed more efficient transferrin attachment and led to significantly enhanced docetaxel efficacy compared to their non-targeted counterparts. Notably, transferrin-bearing PEG5K micelles reduced IC_50_ values by up to 7.3-fold compared to control micelles in PC-3-Luc cells, with similar efficacy improvements observed in DU145 and LNCaP cell lines. This improvement was attributed to enhanced cellular uptake through transferrin receptor-mediated endocytosis, as confirmed by uptake inhibition assays using filipin, chlorpromazine, and free transferrin.

In contrast, PEG10K micelles, despite their advantageous colloidal stability and drug loading capacity, showed reduced transferrin conjugation efficiency and diminished targeting capability. The longer PEG chains likely induced steric hindrance, limiting ligand–receptor interactions and resulting in inferior anti-proliferative activity compared to control micelles. These outcomes highlight the critical balance between PEG-mediated steric shielding and targeting efficiency, where overly long PEG chains may compromise the accessibility of targeting ligands.

Overall, transferrin-conjugated PEG5K zein micelles emerged as a more effective nanocarrier formulation of docetaxel for prostate cancer therapy. The study underscores the importance of rational nanocarrier design, where subtle differences in polymer architecture can markedly influence biological behavior and therapeutic performance.

## Figures and Tables

**Table 1:** Size, polydispersity index, and zeta potential of Tf-bearing and control PEGylated zein micelles entrapping docetaxel (n = 4).

Formulation	Size (nm)	PDI	Zeta Potential (mV)
Tf-PEG5K-zein-DTX	127.7 ± 1.3	0.19 ± 0.01	−24.3 ± 0.9
Tf-PEG10K-zein-DTX	187.5 ± 1.5	0.25 ± 0.01	−12.5 ± 0.8
PEG5K-zein-DTX	100.8 ± 2.8	0.20 ± 0.01	30.7 ± 0.4
PEG10K-zein-DTX	186.5 ± 1.2	0.19 ± 0.00	27.4 ± 1.0

**Table 2:** Anti-proliferative efficacy of Tf-bearing and control PEGylated zein (5 K and 10 K) micelles entrapping docetaxel (control: docetaxel solution) in PC-3-Luc, DU145, and LNCaP prostate cancer cell lines. Results are expressed as mean ± S.E.M. (n = 15). Within each cell line, statistical differences between formulations were evaluated by one-way ANOVA followed by Tukey’s post-test (*p* < 0.05). Statistically significant differences between IC_50_ values are indicated using different superscript letters within each row.

	IC_50_ (µ/mL) (Mean ± S.E.M.)				
	Formulations				
Cell Line	Tf-PEG5K-Zein-DTX	PEG5K-Zein-DTX	Tf-PEG10K-Zein-DTX	PEG10K-Zein-DTX	DTX
PC3	0.09 ± 0.04	0.66 ± 0.14	0.61 ± 0.31	0.28 ± 0.09	0.04 ± 0.01
DU145	0.13 ± 0.05	0.27 ± 0.09	2.67 ± 0.82	1.26 ± 0.28	0.01 ± 0.003
LNCaP	0.04 ± 0.01	0.08 ± 0.03	0.71 ± 0.22	0.04 ± 0.03	0.001 ± 0.0005

## Data Availability

The data that support the findings of this study are available from the corresponding author, [C.D.], upon request.

## Abbreviations

The following abbreviations are used in this manuscript:

ANOVA	One-way analysis of variance
BSA	Bovine serum albumin
CMC	Critical micelle concentration
CPZ	Chlorpromazine
DAPI	4′,6-diamidino-2-phenylindole
DMSO	Dimethyl sulfoxide
DMSO-d_6_	Deuterated dimethyl sulfoxide
D_2_O	Deuterium oxide
DLS	Dynamic light scattering
DTX	Docetaxel
%EE	Percentage of encapsulation efficiency
EPR	Enhanced permeability and retention
FBS	Fetal bovine serum
GRAS	Generally Recognized As Safe
HEPES	4-(2-hydroxyethyl)piperazine-1-ethanesulfonic acid
MEM	Minimum Essential Eagle’s Medium
MFI	Mean fluorescence intensity
mPEG5K-zein	PEGylated zein with a PEG molecular weight of 5000 Da
mPEG10K-zein	PEGylated zein with a PEG molecular weight of 10,000 Da
mPEG-SCM-5K	Methoxy PEG succinimidyl carboxymethyl ester with a PEG molecular weight of 5000 Da
mPEG-SCM-10K	Methoxy PEG succinimidyl carboxymethyl ester with a PEG molecular weight of 10,000 Da
mPEG-zein-DTX	PEGylated zein micelles loading docetaxel
MTT	3-(4,5-dimethylthiazol-2-yl)-2,5-diphenyl-tetrazolium bromide
MWCO	Molecular weight cut-off
PBS	Phosphate buffer saline
PDI	Polydispersity index
PEG	Polyethylene glycol
PLL	Poly-L-lysine
RPMI	Roswell Park Memorial Institute
S.E.M.	Standard error of the mean
TEM	Transmission electron microscopy
Tf	Human holo-transferrin
Tf-mPEG-zein	Transferrin-bearing PEGylated zein
TfR	Transferrin receptor
